# Investigating the effect of geopolitical risk on defense companies’ stock returns

**DOI:** 10.1016/j.heliyon.2024.e40974

**Published:** 2024-12-07

**Authors:** Catalin Gheorghe, Oana Panazan

**Affiliations:** Department of Engineering and Industrial Management, Transilvania University of Brașov, Brașov, Romania

**Keywords:** Defense industry, GPR, Crimean Peninsula annexation, Russia–Ukraine war, Israeli–Hamas conflict

## Abstract

This study examines the influence of geopolitical risk (GPR) on the stock returns of 75 global representative defense companies. Our argument is based on the premise that the Crimean Peninsula's 2014 annexation was a turning point for the defense industry. The study uses wavelet coherence and phase differences to examine daily datasets spanning from January 1, 2014 to December 31, 2023. We find that 50.6 % of the defense companies experienced immediate effects following the Crimean Peninsula's annexation. Among all headquarters, the reaction of companies from Europe, the US, and South Korea in 2014 was noteworthy. Subsequently, during the COVID-19 pandemic, 30.6 % of companies recorded movements with a medium frequency, revealing negative and moderate influence. Furthermore, the Russia–Ukraine war impacted the stocks of 81.4 % of the defense companies in our sample. The results clearly demonstrate the defense industry's global growth trend, particularly after 2014. Additionally, the conflict between Israel and Hamas that began in October 2023 has had a local effect on the Israeli defense industry without contributing to the spread of GPR. The study concludes that investors and decision makers must shift orientation toward knowledge of defense industry stocks to hedge and diversify the risks induced by GPR.

## Highlights

• The Crimean Peninsula's 2014 annexation was a turning point for the defense industry.
• Following the annexation, 50.6 % global defense companies were immediately affected.
• The COVID-19 pandemic had a moderately negative effect on the defense industry.
• The Russia–Ukraine war affected the stocks of 81.4 % of global defense companies.
• The October 2023 Israel–Hamas conflict had a local effect on the defense segment.

## 1 Introduction

This study examines whether changes in the perception of geopolitical risk (GPR) affect global defense companies’ stock prices. To do so, we use the GPR index developed by Caldara and Iacoviello [1] to explore the impact on a cross section of firm-level returns in the defense industry. GPR includes all activities that affect the normal peace process of international relations, including tensions between states or regions, terrorism, elections, nuclear threats, political upheavals, and war. Consequently, according to Caldara and Iacoviello [1], high values of geopolitical tension can facilitate a decline in financial activity, decreased stock returns, and reorientation of fund outflows from emerging countries to more developed nations. Events characterized by political uncertainty and economic instability induce reluctance among investors, which is manifested in postponed investments. Investors subsequently choose assets and markets that are considered to be lower risk. GPR affects asset prices by impacting business decisions, investor behavior, market sentiment, and asset market stability (e.g., Refs. [[2], [3], [4], [5]]).

Stock returns are one of the fundamental indicators in the financial market landscape as they enable market evaluation and serve as a benchmark for informed investment decisions (e.g., Refs. [[6], [7], [8], [9]]). At the company level, GPR influences aspects of profitability [10], bank stability [11], financing decisions [12], corporate investment [13], cash holdings [14], and firms' innovation [15,16]. Shocks that generate GPR can negatively affect defense companies' performance in a specific manner compared with other sectors’ activity.

Defense companies are in a unique position that differs from organizations in other industries that is attributable to strategic importance and technological spin-offs from military to civilian. The defense industry is one of the economic pillars and is vital for national security due to the continuous development of cutting-edge technology [17]. With this particular character, the defense industry supports international security and safety and strengthens peace [18,19]. The commercialization of goods is a serious challenge for the defense industry that involves government officials, foreign governments, and political leaders in addition to defense companies [20,21]. However, great controversies surround the armament trade [22]. In addition, considerable secrecy is traditionally employed in defense spending, which extends to the technologies, materials used, and products obtained. Defense industry programs are executed in compliance with specific regulations and procedures, giving this industry a particular character, justifying the objectives of related studies.

Previous studies have examined the relationship between the military industry and GPR; however, this literature is insufficient. Using data covering the period from November 4, 2009 to March 31, 2014, Yuan et al. [23] employed a generalized autoregressive conditional heteroskedasticity model, determining that China's volatile aerospace and defense industries exhibit asymmetric behavior and that good news induces stronger effects than bad news. Russia's 2022 invasion of Ukraine was a significant event with notable economic, political, and strategic consequences [18]. Defense industry spending increased substantially in 2022 in four of the five world regions according to Tian et al. [24]. According to Yang et al. [25], energy, defense, and tobacco stocks perform better than environmental, social, and governance (ESG) stocks during economic recession. Studies have confirmed the spread of risks in EU markets, which was identified from a dynamic perspective [26], but did not indicate impact on the defense industry. Choi [27] analyzed 11 sectors of the S&P 500 index across three distinct periods that included the 2008 global financial crisis, the COVID-19 pandemic, and the war in Ukraine. Although the author presented conclusive evidence regarding the correlation between the sectors, the defense industry was not highlighted. Zhang et al. [28] conducted large-scale research examining the connection between GPR and the war in Ukraine using a sample of 36 global aerospace and defense companies covering 10 continents between January 2, 2020 and April 4, 2022. Using wavelet analysis, the authors presented novel evidence regarding the connection between GPR, some US companies, and Europe. A similar orientation was also identified by Klein [29], who analyzed 33 aerospace and defense companies in the US, the EU, Canada, and China. Using hierarchical clustering, the author identified four clusters that group companies with similar behavior and the connections between them. Unlike these studies, we investigate a much larger number of defense companies and a longer sampling period.

Moreover, global recovery from the shock generated by the COVID-19 pandemic overlapped with a new period of tension at almost all levels of society and the global economy due to the armed conflict between Russia and Ukraine. In the context of geopolitical tensions and economic sanctions, the effects of the conflict will extend over a much longer period, regardless of the dynamics of the war in Ukraine. According to Fama [6], financial assets are influenced by any information contributing to immediate price adjustments in an efficient market. In this sense, the period under review provides a unique backdrop for understanding the effects of GPR on defense stock returns and the manner in which they evolve globally. We could not identify any studies that examined the specific effects of the COVID-19 pandemic on defense companies.

This study supplements previous research regarding GPR and stock dynamics in the defense industry. First, we consider the annexation of the Crimean Peninsula in 2014 to constitute a revitalization of the defense industry, a trend strengthened by the mutual sanctions initiated by Russia and the NATO states and the war in Ukraine in 2022. Second, this study analyses the influence of GPR on defense industry stock returns from a dynamic perspective, considering the heterogeneous nature of the market. From our perspective, the annexation of the Crimean Peninsula, the war between Russia and Ukraine in 2022, reciprocal sanctions applied by NATO states and Russia, the COVID-19 pandemic, and the Israel–Hamas war have impacted the defense industry globally. The impact of these uncertainties on defense stock returns has not received enough attention. Third, we examine the interdependence between GPRs and markets in terms of time and frequency. Unlike other methods, wavelet analysis allows for the evaluation of lead–lag dynamics between time series, providing information related to market dynamics and how it has been driven by GPRs considered. Time–frequency GPR aggregation provides practical solutions for accurately predicting stock returns.

Our main contributions are summarized as follows. Using time–frequency analysis and a comprehensive set of 75 defense industry companies, we reveal a correlation between GPR and defense stock returns over the analysis period. We accurately detail the dynamic spread and nonlinear risks in defense markets to provide predictive information to help forecast stock defense price returns. Specifically, we provide evidence showing that Russia's annexation of the Crimean Peninsula in 2014 was an impetus for defense companies' repositioning in various countries. We find that this phenomenon intensified in the weeks following the war in Ukraine in February 2022. Compared with other fields of activity, the COVID-19 outbreak had a moderate influence on the defense sector, whereas the Israel–Hamas conflict has only affected Israeli defense companies. The results of this study are related to the dynamics of the defense industry and news accompanying geopolitical events, providing valuable insights for decision makers and investors.

The remainder of this paper is structured as follows. Section 2 presents a synthesis of relevant research on the study's topic. Section 3 details our data and the methodology used. Section 4 outlines the results, section 5 discusses the results, and section 6 presents robustness tests. Finally, section 7 presents our conclusions, study limitations, and future research directions that may expand our results.

## 2 Literature review

In this section, we synthesize the relevant literature focused on the correlation between GPR and defense companies. Regardless of the measurement employed, GPR is a phenomenon that affects all economies. The GPR index is based on news media and assesses the appearance of words that express geopolitical tensions in the online environment. Index construction includes various risks resulting from previous events, referencing Baker et al. [30] and the methodology of Saiz and Simonsohn [31].

This study expands on existing literature by analyzing a broader sample of 75 defense companies over a decade-long period, employing wavelet coherence to capture the dynamic effects of geopolitical risk (GPR) on stock returns. Unlike prior studies focused on isolated events, our approach offers a comprehensive, time-frequency analysis of GPR's impact on the defense sector.

Contemporary digitization has helped reveal the impact of GPR shocks on financial assets. The impact and transmission paths of GPR-generated shocks are an ongoing subject of concern for many researchers worldwide, particularly after the start of war in Ukraine [5,32]. The consequences of GPR include increased costs, higher risks in the financial market [33], and a reduction in investors' interest in risky fund units [34]. For instance, Naeem et al. [35] and Shaik et al. [36] illustrate that geopolitical risks significantly heighten volatility across asset classes, underscoring the importance of analyzing volatility patterns for sectors such as defense, which are highly sensitive to geopolitical changes. GPRs affect economies and have an unfavorable influence on the volatility of natural resources (e.g., Refs. [35,[37], [38], [39], [40]]), stocks (e.g., Refs. [36,[41], [42], [43]]), cryptocurrencies [[44], [45], [46]], gold [8,36], bonds [47,48], and energy [[48], [49], [50]]. As an ongoing event, the war in Ukraine has had a smaller impact on the volatility generated by other events such as the 1987 Black Monday, the 2008 Lehman collapse, and 9/11 attacks, according to Izzeldin et al. [51]. Existing studies have investigated the effects of geopolitical risks on market volatility and risk spillovers, demonstrating how GPR influences the financial market's exposure to higher-order risks (e.g., Refs. [35,36,51]). Building on this foundation, our study uses wavelet coherence to capture both the immediate and dynamic responses of stock returns to GPR over different time horizons.

The end of the Cold War generated a worldwide decline in the defense industry. Under the conditions of the reduced military expenses in budgets, military personnel, and production, defense companies adapted their marketing and diversification strategies [52]. The Cold War's end strengthened the belief that war was unthinkable in the developed world [53,54]. In other words, the probability that consolidated democracies would fight one another was considered to be extremely low [55]. This triggered a considerable decline in research and development (R&D), a trend that affected defense and commercial industries [54]. However, this perspective of a general decline is not fully accepted, as previous research has cited a rise in wars, particularly following the end of the Cold War [56].

The formation and enlargement of the European Union (EU) was a new turning point in the defense industry. Fragmentation of the industry, stagnant budgets, rising costs, size differences among the main US companies, competition from other global manufacturers, export difficulties, and low domestic demand were the primary causes that advanced the industry's slow development [57,58]. The budgets for the European Defence Fund included attempts to stimulate research projects and defense collaboration [59,60]. In recent years, initiatives in the EU have increased security and defense collaboration, including the integration of the defense industry as a key element, in particular by initiating permanent structured cooperation in defense [61,62]. A different reaction of defense companies to GPR events is possible since EU states have different guidelines and security and defense policies regarding integrated defense [63]. We contend that the magnitude of GPR effects differs by region and incurs dynamic disruptive factors for the defense industry in Europe and globally.

Russia's annexation of the Crimean Peninsula was a geopolitical event that occurred in March 2014. After 2014, China expanded its nuclear arsenal, while Russia, the US, the UK, and other states have been developing programs to modernize their arsenals [64]. According to Cheung [65], China's defense system has changed significantly in the last 20 years, largely driven by foreign technology transfers. The number of nuclear warheads has risen, and the development of new anti-ballistic, anti-satellite, and hypersonic weapons is fueling this arms race. This trajectory facilitated increased defense budgets in 2022 (France, 1.94 %; Germany, 1.39 %; UK, 2.3 %; and US, 3 %) [66]. Military spending in 2022 totaled $877 billion in the US, $292 billion in China, and $86.4 billion in Russia [24]. This trend will ensure increased revenue and fuel investments for defense companies in the coming years. Total defense expenditure as a percentage of global GDP remained at 2.2 %, which is one of the minimum values after the Cold War; however, with the increase in global GDP, the budgetary amounts allocated to the military field have increased. The years in which the 2.2 % percentage was exceeded included 2014, 2018, and 2021 [24]. Armament has not yet returned its peak during the Cold War, but the pattern is evident. Defense companies' financing affects stocks' profitability, particularly during GPR crises.

We found financial literature on the subject of Russia's annexation of the Crimean Peninsula to be extremely limited, examining military, political, social, and economic implications. The studies that have investigated economic aspects have focused on sanctions, tariff or trade barriers, limited access to European capital markets, payment systems, asset freezes for individuals or businesses, travel visa rejections, and the retreat of European or Northern American organizations (e.g., Refs. [[67], [68], [69], [70], [71], [72], [73]]). Changes have also been noted in the defense industry of post-communist states. Estonia, Lithuania, and Latvia only produced arms and materials as a function of consumption for the national armed forces, whereas exports from other states such as Czechia, Slovakia, and Hungary were dominated by oligarchs [74]. GPR in recent decades has thrown public security and global economic development out of balance [37].

We include the period between the first appearance of a symptomatic coronavirus case and the World Health Organization's (WHO) declaration of pandemic closure in our study. Numerous studies published in recent years have demonstrated the negative influence of the COVID-19 pandemic on stock returns in various domains [75,76]. Diametrically opposed were the stocks of pharmaceutical companies [77,78], biotechnologies [79,80], and healthcare. Lamba et al. [81] and Piñeiro-Chousa et al. [82] recorded abnormally positive returns, particularly following vaccine development. Investors may have favored defense stocks after the war in Ukraine began, similar to medical companies during the COVID-19 pandemic.

The final GPR in the analysis period is the open conflict between Israel and Hamas, which began on October 7, 2023. Because this conflict started recently, no research was expected to examine its impact on regional financial markets; therefore, we extended our literature search using appropriate words on the research portals. We confirm that no articles have been published on the connection between the GPR and the conflicts in Israel.

While most recent studies have addressed the link between a single event and stock returns, our study adopts a holistic approach, examining multiple high-impact GPR events across a sustained period. This enables us to identify broader, persistent trends in the defense industry's response to geopolitical risk, thus providing deeper insights into its long-term volatility dynamics. Tensions between states and internal conflicts are increasing around the world, raising budgets and promoting the integration of new defense technologies, as also noted by Güneri and Deveci [19]. We contend that the future of defense industry companies will be shaped by emerging threats, technological advances, and European defense policy, as highlighted by Fiott [62]. Given these unique characteristics, our research is more relevant to the defense industry than other sectors. While most existing studies investigate the impact of individual geopolitical events on stock returns, our study adopts a holistic approach, examining multiple high-impact GPR events across a sustained period. This enables us to identify broader, persistent trends in the defense industry's response to geopolitical risk, thus providing deeper insights into its long-term volatility dynamics.

## 3 Data and methodology

### 3.1 Data description and sources

This study addresses the link between GPR and defense industry companies' stock returns. Russia's annexation of the Crimean Peninsula occurred on March 21, 2014. To avoid bias, the starting point of our data is January 1, 2014, and the end point is December 31, 2023, covering the primary GPR events that are the subject of our research. To achieve our research objectives, we collected daily stock exchange values for each company analyzed, resulting in a dataset with 2563 observations per company. Data access was conducted with the consent of the organizations respecting research ethics.

We selected a sample of 75 of the largest companies in the global defense industry, based on the Stockholm International Peace Research Institute (SIPRI) ranking, which is recognized for its objectivity and rigor in collecting data on global defense industry revenues. The SIPRI ranking provides comprehensive sector coverage, ensuring the inclusion of companies that contribute significantly to global revenues and play a strategic role in the industry. This SIPRI-based selection allows for a robust and relevant market analysis, ensuring that our results apply to the most important publicly listed companies in the defense sector. Established in 1966, SIPRI is among the most authoritative and comprehensive open-source databases on defense expenditure, covering 174 countries from 1949 to 2022 [83]. We identified the top 75 companies listed in various markets from this ranking, excluding companies for which the necessary information for our analysis was not identified. The distribution of companies by country is as follows: USA, 32 companies; China and the UK, 6 companies; France, 5 companies; Japan and South Korea, 4 companies; Germany, India, and Israel, 3 companies; Italy, 2 companies; and Australia, Canada, Norway, Poland, Singapore, Sweden, and Turkey, 1 company.^1^

We compiled a time series comprising the daily returns in the analyzed period for each company, obtaining the daily data from the Investing platform [84]. Table 1 presents the analyzed companies and the symbol of the company in the SIPRI ranking. We compare companies’ daily stock returns using the GPR index created by Caldara and Iacoviello [1]. We obtained the daily GPR index data from the Matteo Iacoviello platform [85]. Appendix 1 presents the descriptive statistics, and Appendix 2 illustrates the graphic trends of each time series. We calculate daily yield applying the relationship Rt=ln(PtPt−1)∙100, where P_t−1_ denotes the stock price at period t − 1, P_t_ represents stock price in period t, and R_t_ represents stock price return in period t [86]. We used EViews 13 (Quantitative Micro Software, USA) and MATLAB version 9.13.0 (R2023b) to process our time series data.

**Table 1:** Defense industry companies analyzed.

Rank	Company	Symbol	Country	Arms revenue in 2022 (million USD)
1	Lockheed Martin Co.	LMT	US	59,390
2	Raytheon Technologies Co., BDR	RYTT34	US	39,570
3	Northrop Grumman Co.	NOC	US	32,300
4	Boeing Co.	BA	US	29,300
5	General Dynamics Co.	GD	US	28,320
6	BAE Systems PLC	BAES	UK	26,900
7	NORINCO International Cooperation Ltd.	000065	China	22,060
8	AVIC Aircraft Co., Ltd.	000768	China	20,620
9	China Aerospace Times Electronics Co., Ltd.	600879	China	19,560
10	CETC Cyberspace Security Technology Co., Ltd.	002268	China	15,080
11	L3Harris Technologies, Inc.	LHX	US	12,630
12	Leonardo SpA	LDOF	Italy	12,470
13	Airbus Group SE	AIR	France	12,090
14	CSSC Offshore & Marine Engineering Group, Ltd.	600685	China	10,440
15	Thales Group	TCFP	France	9420
16	Huntington Ingalls Industries, Inc.	HII	US	8750
17	Leidos Holdings, Inc.	LDOS	US	8240
18	Booz Allen Hamilton Holding	BAH	US	5900
19	Dassault Aviation SA	AM	France	5070
20	Elbit Systems, Ltd.	ESLT	Israel	4960
21	Rolls–Royce Holdings PLC	RR	UK	4930
22	CACI International, Inc.	CACI	US	4820
23	Honeywell International, Inc.	HON	US	4630
24	Rheinmettal AG	RHMG	Germany	4550
25	General Electric Company	GE	US	4410
26	KBR, Inc.	KBR	US	4270
27	Safran SA Company	SAF	France	4200
28	Israel Aerospace Industries, Ltd.	ILARSP4 = TA	Israel	4100
29	Science Applications International	SAIC	US	3780
30	SAAB	SAABBs	Sweden	3700
31	Babcock International Group PLC	BAB	UK	3680
32	Hindustan Aeronautics, Ltd.	HIAE	India	3460
33	Rafael Holdings, Inc.	RFL	Israel	3380
34	Mitsubishi Heavy Industries, Ltd.	7011	Japan	3250
35	Textron, Inc.	TXT	US	2910
36	Fincantieri SpA	FCT	Italy	2820
37	CEA Industries, Inc.	CEAD	France	2790
38	Hanwha Aerospace Co., Ltd.	012450	South Korea	2780
39	V2X, Inc.	VVX	US	2520
40	Transdigm Group, Inc.	TDG	US	2330
41	Parker–Hannifin Co.	PH	US	2270
42	Singapore Tech Engineering, Ltd.	STEG	Singapore	2180
43	Oshkosh Co.	OSK	US	2140
44	Jacobs Engineering Group, Inc.	J	US	2090
45	Teledyne Technologies, Inc.	TDY	US	2020
46	Aselsan Elektronik Sanayi ve Ticaret AS	ASELS	Türkiye	2020
47	CNNC International, Ltd.	2302	China	1940
48	Thyssenkrupp AG	TKAG	Germany	1930
49	Bharat Electronics, Ltd.	BAJE	India	1920
50	Serco Group	SRP	UK	1850
51	Kawasaki Heavy Industries, Ltd.	7012	Japan	1830
52	LIG Nex1 Co., Ltd.	079550	South Korea	1720
53	BWX Technologies, Inc.	BWXT	US	1700
54	Hensoldt Ag	HAGG	Germany	1660
55	Qinetiq Group PLC	QQ	UK	1620
56	Pan Global Resources, Inc.	PGZ	Poland	1600
57	Korea Aerospace	047810	South Korea	1550
58	Parsons Co.	PSN	US	1540
59	Eaton Co. PLC	ETN	US	1520
60	CAE, Inc.	CAE	Canada	1420
61	Curtiss–Wright Co.	CW	US	1390
62	Moog, Inc.	MOGa	US	1280
63	Fujitsu General Ltd.	6755	Japan	1270
64	Kongsberg Gruppen ASA	KOG	Norway	1230
65	Amphenol Co.	APH	US	1140
66	Melrose Industries PLC	MRON	UK	1060
67	Mazagon Dock Shipbuilders, Ltd.	MAZG	India	1000
68	Austal, Ltd.	ASB	Australia	980
69	Mercury Systems, Inc.	MRCY	US	960
70	Ball Co.	BALL	US	930
71	Howmet Aerospace, Inc.	HWM	US	920
72	TTM Technologies, Inc.	TTMI	US	860
73	Heico Co.	HEI	US	860
74	Hyundai–Rotem	064350	South Korea	820
75	IHI Co.	7013	Japan	790

Note: This table lists the top 75 defense companies worldwide, as ranked by the Stockholm International Peace Research Institute (SIPRI). Source: Author's compilation based on SIPRI data for 2022 arms revenue.

### 3.2 Methodology

We chose our processing method according to the research objectives pursued and advantages compared with other techniques, selecting wavelet analysis to process our time series because of its advantages over traditional regression approaches. For example, Bouri et al. [9] stated that investors can adopt portfolio modeling decisions and strategies over different investment time horizons using wavelet analyses. According to Qiao et al. [87] to obtain the best returns, investors may take short- or long-term positions in the market [87]. Haq et al. [88] argues that this approach captures the relationship between two time series in terms of time and frequency more accurately via bivariate wavelet coherence [88]. Given these advantages, this method is suitable for examining the interactions between GPR and stock returns series compared to classical statistical methods.

Recent studies have employed continuous wavelet transform to capture GPR in financial time series (e.g., Refs. [28,42,45,46,89]). ψ(t) is a function that meets the requirements $\int_{‐\infty }^{+\infty }\psi \left(t\right)\text{dt}=0$ and $\int_{‐\infty }^{+\infty }{\left|\psi \left(t\right)\right|}^{2}\text{dt}\;<\infty$, called the “mother” wavelet. From this, we obtain a family of functions called “daughter” wavelets $\psi_{\tau ,s}\left(t\right)$ denoted by translation and scaling on the time axis as follows:

(1) $$\psi_{\tau ,s}\left(t\right)=\frac{1}{\sqrt{\left|s\right|}}\psi \left(\frac{t-\tau }{s}\right)s,\tau \in R,s\neq 0\text{.}$$

where τ∈R is a location parameter, and s≠0 is a scale parameter so that $\psi \left(\tau \right)=\psi \left(t\;–\;\tau \right),\psi \left(s\right)=\psi \left(\frac{t}{s}\right)$. The scaled and translated wavelet has the following form $\psi_{\tau ,s}\left(t\right)=\psi \left(\frac{t-\tau }{s}\right)\text{.}$ As shown in Equation (1), this formulation adapts the wavelet to the time-frequency characteristics of the analyzed series.

By moving and scaling the wavelet function along time series x(t), we reveal an overlap called convolution, which is expressed as follows:

(2) $$W_{x}\left(\tau ,s\right)=\frac{1}{\sqrt{\left|s\right|}}\int_{‐\infty }^{\infty }x\left(t\right)\Psi^{*}\left(\frac{t-\tau }{s}\right)\text{dt.}$$

where ∗ denotes the conjugated complex form. Equation (2) represents the continuous wavelet transform of x(t), which highlights both time and frequency domain features.

Among the types of waves designed in the literature over time, we apply a continuous Morlet wavelet, which has been frequently used in time series [42,46] modeling, and is defined as follows:

(3) $$\psi^{M}\left(t\right)=\pi^{-\frac{1}{4}}\;{∙e}^{i{∙\omega }_{0}∙t}∙e^{-\frac{t^{2}}{2}}\text{,}$$

where ω_0_ is a wavelet center frequency and $e^{i{∙\omega }_{0}∙t}$ is the wavelet function's imaginary section at point (0, ω_0_/2 · π). As defined in Equation (3), the Morlet wavelet is widely recognized for its ability to provide localized frequency information while maintaining high temporal resolution.

Between frequency f and scaling factor s we use the following:

(4) $$f=\frac{\mu_{f}}{s}\approx \frac{1}{s}\text{.}$$

Equation (4) establishes the inverse proportionality between frequency and the scale factor, which is critical in translating wavelet scale to the corresponding frequency domain. To ensure admissibility, we selected a value of w_0_ greater than 5 for the Morlet wavelet, as suggested in the literature [90,91]. For values of w_0_ > 5, the correction term becomes negligible, which allows for reliable time-frequency analysis without significant distortions in wavelet coefficients.

This study uses the following wavelet power spectrum (WPS) to evaluate the magnitude of volatility on the time dimensions:

(5) $${\text{WPS}}_{x\left(y\right)}={\left\lceil W_{x\left(y\right)}\left(\tau ,s\right)\right\rceil }^{2}$$

As shown in Equation (5) the WPS quantifies the energy distribution over time and frequency, providing insights into the temporal dynamics of volatility.

In the space of two time series, x(t) and y(t), the wavelet coherence is defined as follows:

(6) $$R_{x,y}^{2}\left(\tau ,s\right)=\frac{{\left\lceil S\left(s^{-1}W_{x_{i}y_{j}}\left(\tau ,s\right)\right)\right\rceil }^{2}}{S\left(s^{-1}{\left|W_{x_{i}}\left(\tau ,s\right)\right|}^{2}\right)\;*\;S\left(s^{-1}{\left|W_{x_{j}}\left(\tau ,s\right)\right|}^{2}\right)},{R_{x,y}}^{2}\in \left[0,1\right]$$

where W_xi_ and W_yj_ are the wavelet transformations of x and y, and W_xixj_ is the wavelet cross-spectrum between x and y, respectively. Equation (6) measures the local correlation between the series in the time-frequency domain.

## 4 Results

As noted above, Appendix 1 presents the descriptive statistics of the defense industry stock returns. The data presented reveals that the analyzed time series do not follow a normal distribution as nonzero skewness values were identified for all series. The mean values show variability across companies, indicating differences in average performance. Standard deviation highlights return dispersion, with higher values for some companies pointing to increased volatility, potentially linked to GPR events.

Additionally, the inter-quartile range (IQR) was calculated for each series, as shown in Appendix 1, providing a robust measure of dispersion that is less sensitive to outliers compared to standard deviation. Higher IQR values, such as those for CEAD (0.06373) and RFL (0.04285), indicate greater variability in the central range of returns, suggesting increased sensitivity to geopolitical events. In contrast, companies like ILARSP4 = TA (0.00079) and PGZ (0.00000) show lower IQR values, indicating lower variability and potentially more stable behavior within the interquartile range. This additional measure enhances the reliability of our analysis by accurately representing the typical spread of returns and highlighting differences in volatility profiles among companies.

Skewness values show asymmetry in returns: negative skewness for some companies suggests more frequent extreme negative returns, while positive skewness for others indicates occasional positive extremes. Kurtosis values above 3 confirm leptokurtic distributions, implying a higher probability of extreme values. Together, these moments provide insights into the defense stocks' distributional properties, suggesting GPR's influence on market behavior.

To ensure the robustness of our analysis, we conducted Zivot-Andrews, Dickey-Fuller, and Phillips-Perron (PP) tests to confirm the stationarity of the return series, accounting for potential structural breaks (Appendix 6). The Zivot-Andrew's test results indicate that a considerable number of stock return series remain non-stationary when structural breaks are included, reinforcing the idea that significant geopolitical risk (GPR) events cause enduring shifts in defense stock behavior. Stocks such as RYTT34 and NOC, among others, show t-statistics that do not reject the null hypothesis of non-stationarity, signaling potential long-term instability in the defense sector under GPR influence. In contrast, the Dickey-Fuller and Phillips-Perron tests generally confirm stationarity in the absence of structural breaks, highlighting the importance of these breaks to fully understand GPR's impact on defense stocks. Additionally, ARCH effect tests confirm significant conditional heteroskedasticity across many series, indicating that defense stocks experience varying levels of volatility in response to GPR events. These results validate the application of wavelet analysis, allowing us to explore the time-frequency dynamics of volatility and co-movement in defense stocks under geopolitical pressures, providing nuanced insights into the sector's risk profile over different time horizons.

In accordance with this study's objectives, we created two pairs of temporary series for each defense company analyzed, with each pair including the GPR index and the daily stock return. Appendix 3 presents a contour map incorporating wavelet coherence phase difference and wavelet coherence (WCT) for each company under analysis. Such plots have been frequently used to examine the relationship between volatility or stock returns and GPR (e.g., Refs. [35,39,45,46,89,[92], [93], [94]]). The colors in each graph indicate the intensity of the phase difference between dark blue (minimum values) and dark yellow (maximum). In the scalograms, yellow and blue denote regions with strong and weak movement. Intermittent yellow areas are observed at the top (short term) and bottom (long term) of each scalogram, and yellow regions are encountered over specific periods.

Each graph in Appendix 3 exhibits eight one-way arrows. The x-axis represents time in days, and the y-axis denotes the normalized frequency as a coefficient between 0 and 1. When the time series have a positive correlation, the arrows point to the right (in phase), while the arrows pointing to the left indicate that the series are negatively correlated (out of phase). Right-pointing upward arrows (↗) or left and downward arrows (↙) confirm that the first variable (GPR) has the dominant role. Downward arrows pointing right (↘) or left and up (↖) indicate the impact of the second variable role (stock returns). Arrows ↑ (↓) indicate a phase shift of π/2 between the two analyzed series.

## 5 Discussion

The results confirm that the GPR events included significantly affected stock returns in the defense industry, with heterogeneous and time-varying influence. The scalograms in Appendix 3 demonstrate that Russia's annexation of the Crimean Peninsula in 2014 is correlated with 29 companies' the reaction in the early weeks, on the low frequency values in the following states: US (NOC, GD, BAE, LHX, HII, LDOS, AM, HON, KBR, VVX, J, TDY, BWXT, TTMI), South Korea (012450, 079550, 047810, 064350), France (AIR 4, TCFP, CEAD), Israel (ILARSP4 = TA), UK (MRON), Australia (ASB), China (000065), Germany (RHMG), Italy (FCT), Norway (KOG), and Poland (PGZ). In addition, nine companies exhibited medium frequency values, including the US (TDG, PH, OSK, PSN, and ETN), UK (SRP and QQ), Germany (TKAG), and Canada (CAE). Notably, 50.6 % of the companies had an immediate reaction to the peninsula annexation in 2014. The reaction of companies from Europe is particularly notable, followed by companies from the US and South Korea. These results lead us to conclude that Russia's annexation of the Crimean Peninsula triggered a relaunch in the defense industries in several nations. In our opinion, the results cannot be attributed to the UN's adoption of a controversial Arms Trade Treaty in April 2013 [95]; therefore, we can extend the conclusions drawn by Yousaf et al. [96] to companies in the defense industry. The findings empirically demonstrate that the invasion of Russian troops had a strong negative impact on most of the stock markets in G20 countries.

Another notable observation is the permanent exit (on low frequency values) of 11 companies, of which 8 were from the US (NOC, LHX, CACI, HON, TXT, TDG, ETN, and PSN), 2 from Germany (RHMG and HAGG), and 1 from Canada (CAE). This result confirms the relaunch of US and German defense companies following Russia's annexation of the Crimean Peninsula in 2014. To confirm these results, we extend the analysis to all companies that recorded continuous comovement in low frequency values, identifying companies that had continuous (long term) comovement until the end of the analysis period after 200 days (TKAG), 400 days (RFL), 500 days (BALL), 600 days (APH, RR, and BAB), 700 days (LMT, BA, CEAD, J, and PH), 800 days (ASB and HIAE), 900 days (OSK, 6755, and SSAB), 1000 days (HII, GE, MOG, and MRON), 1100 days (AIR, ESLT, STEG, and TDY), 1200 days (000065, 002268), 1300 days (BAES), 1400 (ILARSP4 = TA), and 1500 days (GD). Continuous comovement is most notable for some large companies in the US and China, and a similar reaction is evident for the main companies in France, Germany, and the UK.

The pandemic period between March 11, 2020 [97], until the WHO declaration of its closure on May 5, 2023 [98] triggered reactions from 23 companies (30.6 %), on medium frequency values, revealing impact on companies from the USA (LMT, LHX, PSN), UK (BAES, QQ), China (600879, 002268), France (AM, 600685), Germany (RHMG), and Norway (KOG). Other companies only reacted in the second half of the pandemic, including the US (GD, BAH), China (000768), Israel (ESLT), Sweden (SAABBs), and India (MAZG). Other weak reactions can also be observed in the second part of the pandemic, as follows: US (KBR, TDY, MRCY), Italy (LDOF), Israel (ILARSP4 = TA), France (CEAD). The pandemic's influence on defense companies' stocks was negative and moderate compared with other severely affected areas of activity. We find an inverse evolution of the stocks in the defense industry compared with the results presented by Yousaf et al. [99]. Although the authors concluded that in the long term, the effect of the Russia–Ukraine war was weaker compared with the impact of COVID-19, our findings show an inverse dynamic in the defense sector over the same time horizon.

The start of the military conflict between Russia and Ukraine caused strong comovement for all companies, on low and medium frequency values, less US (RYTT34, SAIC, BWXT, TTMI, HEI), Japan (7011, 7012, 6755), China (600685, 2302), South Korea (012450), Singapore (STEG), India (BAJE), and Poland (PGZ). The proportion of companies that had not reacted after February 2022 was 14/75 = 18.6 %. Echoing the findings in 2014, geographical distance may explain the lack of reaction of some companies in the US, Japan, South Korea, and Singapore. The Polish company (the only one in Eastern Europe) did not react to the Russian invasion and represents an exception to the previous conclusion.

The defense decisions adopted in the EU after the start of the Ukrainian conflict triggered market responses. Chovančík and Krpec [100] demonstrated that the war had major implications for European defense industries in general, which is confirmed by our results. We can also anticipate that massive investments in this sector after the beginning of the conflict facilitated the appreciation of these stocks and attracted investors’ attention.

Since 2016, the EU has committed to altering the mechanisms by which defense industrial cooperation is financed and encouraged [61,62]. This turning point may explain the arrow clouds identified on some defense company scalograms in the EU. After the start of the conflict in Ukraine, the EU altered processes related to procurement and joint programs in the field of defense that can explain the increased yield for many of the investigated companies [59,101]. Some highly relevant mergers and acquisitions examined by Kleczka et al. [57] may explain the clouds of points on the company scalograms at different frequency values. Germany, France, and the UK have technologically advanced defense industries and can be engines for consolidating the EU's defense and the development of a transnational defense industry. The evolution of the companies in these states exhibits some similarities that can be explained by the economic relationships between the states, common policies toward Russia during the analysis period, and NATO membership. We attribute the results to geographic proximity to the war zone, similar to Kumari et al. [102]. Another explanation for some of the yellow areas at the top of the scalograms (short-term) may be the impact of the sanctions between NATO states and Russia that occurred in 2014 and after the outbreak of the war in 2022. Our results complement the evidence presented by Klein [29], who observed clustering of US and EU defense companies and a positive correlation between them that intensified after the start of the war in Ukraine. Considering the much larger number of companies analyzed in this study, we can confirm the hypothesis advanced by the author in that study, which proposed that only a portion of defense companies' stocks reacted to the arrival of the news.

The war between Israel and Hamas that broke out on October 7, 2023 can explain the dynamics of Israeli defense companies’ stocks. A weak link between GPR and the stocks of these companies is found in low frequency values. A more pronounced influence is evident for the RFL company scalogram.

Comparative analysis of the scalograms related to the states in the first positions compared with those at the bottom in the ranking does not enable us to draw any conclusions related to companies' size. Therefore, we cannot advance the conjecture concerning differentiated evolution depending on companies’ ranking position, although global military spending is highly concentrated among the most developed companies.

Increased defense industry stock returns are particularly evident in 2022 and partially in 2014, which allows us to confirm and complement the increased interest in the aerospace and defense sectors demonstrated by Singh et al. [46]. Overall, GPR events drive stock returns for most defense companies. Moreover, we consider the evolution of the defense industry as a global phenomenon, confirming the “flight-to-arms” phenomenon that was demonstrated by Zheng et al. [49].

The particular character of defense companies is that other variables may cause deviations in the financial market such as impediments to effective supervision, limited transparency, the involvement of political decision makers, and corruption that are not characteristic of other industrial sectors [21]. In a period dominated by heterogeneous GPR events, investors’ expectations could change, shaping their decisions on the defense industry.

Our results have implications for both investors and policymakers. The significant positive comovement between GPR and long-term defense stock returns suggests limited gains from diversification, positioning defense stocks as key assets for building GPR-adjusted portfolios. Portfolio managers can use these findings to rebalance portfolios based on bullish, normal, and bearish market conditions, aligning investment horizons with the varying impact of GPR. For policymakers, these insights support the development of regulations to mitigate short-selling and panic selling during heightened GPR, helping to stabilize market perceptions. By using wavelet analysis to capture the dynamic interactions between GPR and defense stocks, our study provides actionable insights at both the macro and micro levels, aiding investors in risk-adjusted portfolio construction and policymakers in stabilizing market conditions.

The ties between firms within the same state, the mode of financing within military blocs, firms’ participation in mergers and acquisitions, international defense-related tenders, multinational military aircraft programs, joint defense R&D and production capabilities, and dependence on external suppliers are the main considerations of our robustness tests.

## 6 Robustness tests

### 6.1 ADF results

We use an augmented Dickey–Fuller (ADF) statistic to verify that no stationary cointegration exists among the components of a p-dimensional cointegrated I_(2)_ vector of measurable δ_t_ [103]. Since 0 < σ < s, we infer that the cointegrating rank of r_t_ equals σ. Under the null hypothesis, the following model generates r_t_:

(7) $$\left(\begin{array}{cc} I_{r} & \beta \\ 0 & I_{p-r} \end{array}\right)\left(\delta_{t}-\tau_{t}\right)=\gamma_{t},\left(\begin{array}{cc} \Delta I_{r} & 0\\ 0 & \Delta^{2}I_{p-r} \end{array}\right)\gamma_{t}=\varepsilon_{t}$$

Equation (7) represents the cointegrated model where β is a r x (p – r) matrix, $\varepsilon_{t}$ is a zero-mean, I_(0)_ is a vector process with a finite and nonsingular spectral density at all frequencies, and $I_{s}$ is the s-row identity matrix. Additionally, $\tau_{t}$ integrates deterministic terms for any p-dimensional random vector $V_{t},V_{\left(1\right)t}$, becoming the vector that includes the first r elements of $V_{t}$, and $V_{\left(2\right)t}$ integrates the remaining components, resulting in $V_{t}={\left(V_{\left(1\right)t}^{\prime },V_{\left(2\right)t}^{\prime }\right)}^{\prime }$. Therefore, (x_1_) can also be expressed as follows:

(8) $$\delta_{\left(1\right)t}+\beta_{\left(2\right)t}=\tau_{\left(1\right)t}+{\beta \tau }_{\left(2\right)t}+\gamma_{\left(1\right)t}\;\delta_{\left(2\right)t}=\tau_{\left(2\right)t}+\gamma_{\left(2\right)t}$$

Equation (8) shows that the distinct elements $I_{\left(2\right)}$ and $\delta_{\left(2\right)t}$ do not cointegrate.

Appendix 4 presents the results of our unit root tests, which confirm that all series are stationary and do not follow a stochastic process. The daily log returns are significant at 1 %, 5 %, and 10 % levels, similar to the findings of Phiri et al. [93].

### 6.2 Cross-Wavelet Transform (XWT)

Cross-Wavelet Transform (XWT) allows the identification of the similarity between two time series [104,105] as it identifies the phase relation and areas of high common power [106]. The XWT of time series x(t) and y(t) are:

(9) $$W_{xy}\left(a,b\right)=W_{x}\left(a,b\right)W_{y}^{*}\left(a,b\right)$$

where $W_{x}\left(a,b\right)$ and $W_{y}\left(a,b\right)$ are the wavelet transforms of series x(t) and y(t). Equation (9) measures the cross-wavelet amplitude, $\left|W_{xy}\left(a,b\right)\right|$, measures the coherence magnitude between the two time series at a given scale and point in time. The cross-wavelet phase arg $\left(W_{xy}\left(a,b\right)\right)$ indicates the phase difference of the time series.

Appendix 5 presents the results of XWT application. The spectrograms indicate the correlation of the series in frequency and time coordinates. The findings reveal that most of the series in the high frequency area are highly correlated, indicating a link between short-term variables. A possible explanation for this finding is that financial markets strive to maintain balance to reduce the impact of external factors [107].

In the majority of series, frequency bands colored in blue-green are observed, indicating a consistent but variable correlation over the time series. Returns are continuously influenced by geopolitical risks with impacts that vary in intensity, indicating the existence of a bidirectional relationship between the two series over long horizons.

Similar to Kang et al. [108], increased contagion emerges at certain time periods. The XWT results indicate a relatively high degree of commotion, variations in amplitude, and frequency over the sampled period, for RYTT34 (2017–2019); 000065, 600879, and 002268 (2015–2016); 600685 (2017–2019 and 2017–2018); PGZ (2015–2017), and 2302 (2020–2022). In those periods, geopolitical risks significantly impacted returns. In these series, the spectrograms show almost non-existent correlation areas outside the identified periods, suggesting a variable influence of GPR.

In conclusion, while the CWT scalograms highlight the individual strength of time series at various frequencies [109] and highlight significant oscillations, the XWT spectrograms indicate regions where time series are correlated, providing a detailed dynamic overview of the relationship between defense industry stock returns and geopolitical risks.

## 7 Conclusions

This study investigates the relationship between GPR and stock returns in the global defense industry. We demonstrate how tradeoffs between GPR and stock returns and their volatilities vary with time and scale employing the WCT technique. We analyze the relationships between 75 defense stocks and GPR using daily returns and GPR index data from 2014 to 2023. XWT and ADF tests corroborate the robustness of our findings.

We determine that Russia's annexation of the Crimean Peninsula triggered defense companies' reactions. The results reveal notable lead–lag patterns at various frequencies, time scales, and differences between GPRs and stock returns. In times of financial instability such as the invasion of Crimea in 2014, the COVID-19 pandemic, and the ongoing conflict in Ukraine, a substantial correlation between GPR and stock performance typically occurs. The pandemic period caused the reaction of 23 companies in medium frequency bands, whereas another five companies only reacted in the second part of the pandemic. The influence of the pandemic was weaker for defense companies than that in other fields of activity. The influence of the Israel–Hamas conflict from October 2023 is weak and exhibits a local effect.

The findings of this study can be referenced by market stakeholders, investors, and policymakers as they provide valuable insights into portfolio modeling and effective risk management strategies. Therefore, hedging or diversification decisions can be substantiated upon based on the occurrence of GPR events for each defense company included in our study. We also offer decision-making options for macro-prudential authorities concerning budget allocations to defense and adjusting security policies.

It is natural that although our research offers an accurate review of the influence of GPR on the defense industry over a relevant period, it has certain limitations. One of these concerns the 25 companies for which we were unable to locate stock prices. Another limitation is the absence of companies from other areas of the world such as Russia, the Middle East, and Eastern Europe. Using the limits identified in this study as a starting point, future research directions can be outlined. In this regard, given that military spending affects nations' health spending, a comparative analysis of the different dynamics between medical and defense companies' stocks would be useful. Finally, additional research to consider the different tock returns of companies that produce military aircraft, drones, and other products in the defense industry such as R&D companies would be insightful. Additionally, future studies could employ alternative approaches to wavelet analysis, such as a methodology that defines wavelets by full width at half maximum (FWHM) rather than cycle count, potentially offering enhanced interpretability in time-frequency dynamics. While this study utilizes the general GPR index, future research could incorporate the GPR sub-indices (Acts and Threats) to capture more specific dimensions of geopolitical risk. Analyzing these sub-indices may provide a deeper understanding of how distinct types of geopolitical events affect stock returns in the defense sector. Furthermore, employing Inter-Trial Phase Coherence (ITPC) across key geopolitical events could reveal periods of synchronized market responses, offering additional insights into the defense sector's collective reaction to heightened geopolitical tensions.

## CRediT authorship contribution statement

**Catalin Gheorghe:** Writing – review & editing, Writing – original draft, Visualization, Validation, Supervision, Software, Resources, Project administration, Methodology, Investigation, Funding acquisition, Formal analysis, Data curation, Conceptualization. **Oana Panazan:** Writing – review & editing, Writing – original draft, Visualization, Validation, Supervision, Software, Resources, Project administration, Methodology, Investigation, Funding acquisition, Formal analysis, Data curation, Conceptualization.

## Ethical approval statement

Not applicable.

## Data availability statement

The datasets used and/or analyzed during the current study are available from the corresponding author on reasonable request.

## Funding

This research did not receive any specific funding.

## Declaration of competing interest

The authors declare that they have no known competing financial interests or personal relationships that could have appeared to influence the work reported in this paper.

## Footnotes

Appendix A Supplementary data to this article can be found online at https://doi.org/10.1016/j.heliyon.2024.e40974.

## Footnotes

1 The code represents the company symbol on the https://www.investing.com platform.
