# Supplementary material for: Investigating the effect of geopolitical risk on defense companies’ stock returns
Source: Heliyon. 2024 Dec 7;10(24):e40974. doi: 10.1016/j.heliyon.2024.e40974 (PMC11700249; doi:10.1016/j.heliyon.2024.e40974)
Supplement: Multimedia component 1 [file mmc1.docx]

Appendix 1

Descriptive statistics of the returns

|  | Mean | Median | Maximum | Minimum | Std. Dev. | Skewness | Kurtosis | Proba-  bility | IQR |
| --- | --- | --- | --- | --- | --- | --- | --- | --- | --- |
| LMT | 0.00043 | 0.00044 | 0.10191 | -0.13653 | 0.01396 | -0.59453 | 18.5611 | 0.0000 | 0.01248 |
| RYTT34 | 0.00039 | 0.00000 | 0.34944 | -0.29370 | 0.02073 | 1.07897 | 72.5510 | 0.0000 | 0.00000 |
| NOC | 0.00055 | 0.00037 | 0.12223 | -0.10699 | 0.01514 | -0.02216 | 11.3412 | 0.0000 | 0.01402 |
| BA | 0.00026 | 0.00009 | 0.21768 | -0.27244 | 0.02458 | -0.52296 | 22.9379 | 0.0000 | 0.01960 |
| GD | 0.00039 | 0.00054 | 0.08753 | -0.11571 | 0.01390 | -0.37883 | 9.98632 | 0.0000 | 0.01392 |
| BAES | 0.00036 | 0.00045 | 0.10008 | -0.08701 | 0.01435 | -0.12905 | 8.36166 | 0.0000 | 0.01450 |
| 000065 | 0.00000 | 0.00000 | 0.10283 | -0.51092 | 0.03196 | -2.33362 | 39.6860 | 0.0000 | 0.02443 |
| 000768 | 0.00033 | 0.00000 | 0.11227 | -0.10556 | 0.02789 | -0.09287 | 6.36344 | 0.0000 | 0.02473 |
| 600879 | -0.00008 | 0.00000 | 0.09596 | -0.79896 | 0.02776 | -9.46905 | 271.128 | 0.0000 | 0.02106 |
| 002268 | 0.00016 | 0.00000 | 0.09565 | -0.57536 | 0.03280 | -2.10679 | 41.0447 | 0.0000 | 0.02829 |
| LHX | 0.00043 | 0.00025 | 0.11215 | -0.15431 | 0.01578 | -0.16772 | 14.5317 | 0.0000 | 0.01457 |
| LDOF | 0.00039 | 0.00047 | 0.15072 | -0.25039 | 0.02324 | -0.78090 | 16.7939 | 0.0000 | 0.02324 |
| AIR | 0.00036 | 0.00061 | 0.18618 | -0.25073 | 0.02222 | -0.45116 | 19.1527 | 0.0000 | 0.02044 |
| 600685 | 0.00015 | 0.00000 | 0.09554 | -0.15542 | 0.03025 | 0.01718 | 6.22931 | 0.0000 | 0.02422 |
| TCPF | 0.00041 | 0.00006 | 0.12854 | -0.11947 | 0.01605 | 0.12265 | 10.3890 | 0.0000 | 0.01554 |
| HII | 0.00041 | 0.00065 | 0.10809 | -0.13112 | 0.01711 | -0.39327 | 9.55311 | 0.0000 | 0.01693 |
| LDOS | 0.00045 | 0.00055 | 0.10231 | -0.20358 | 0.01744 | -1.38096 | 21.2128 | 0.0000 | 0.01604 |
| BAH | 0.00078 | 0.00090 | 0.09693 | -0.20938 | 0.01613 | -1.42025 | 21.5463 | 0.0000 | 0.01521 |
| AM | 0.00026 | 0.00000 | 0.11521 | -0.12134 | 0.01805 | -0.04861 | 8.26311 | 0.0000 | 0.01759 |
| ESLT | 0.00049 | 0.00000 | 0.14431 | -0.11162 | 0.01622 | -0.10686 | 9.40221 | 0.0000 | 0.01655 |
| RR | -0.00055 | 0.00000 | 0.36298 | -1.08973 | 0.03657 | -9.81547 | 316.543 | 0.0000 | 0.02213 |
| CACI | 0.00058 | 0.00028 | 0.14011 | -0.14921 | 0.01657 | -0.15093 | 14.6456 | 0.0000 | 0.01570 |
| HON | 0.00032 | 0.00047 | 0.14036 | -0.12882 | 0.01411 | -0.19895 | 15.6053 | 0.0000 | 0.01264 |
| RHMG | 0.00072 | 0.00070 | 0.22155 | -0.12545 | 0.02186 | 0.49608 | 12.6867 | 0.0000 | 0.02164 |
| GE | -0.00011 | 0.00000 | 0.13746 | -0.16441 | 0.02120 | -0.11217 | 10.3272 | 0.0000 | 0.01763 |
| KBR | 0.00021 | 0.00000 | 0.16821 | -0.26000 | 0.02396 | -1.08751 | 19.4144 | 0.0000 | 0.02171 |
| SAF | 0.00045 | 0.00032 | 0.19006 | -0.25973 | 0.02067 | -0.69034 | 27.4478 | 0.0000 | 0.01790 |
| ILARSP4=TA | 0.00000 | 0.00000 | 0.02273 | -0.02897 | 0.00205 | -3.02306 | 67.1338 | 0.0000 | 0.00079 |
| SAIC | 0.00052 | 0.00070 | 0.17177 | -0.20109 | 0.01936 | -1.34150 | 22.2555 | 0.0000 | 0.01737 |
| SAABBs | 0.00049 | 0.00000 | 0.14237 | -0.14532 | 0.01910 | -0.09068 | 12.4155 | 0.0000 | 0.01689 |
| BAB | -0.00043 | 0.00000 | 0.27751 | -0.17906 | 0.02125 | 0.45280 | 20.8646 | 0.0000 | 0.01949 |
| HIAE | 0.00109 | 0.00000 | 0.15940 | -0.15274 | 0.02221 | 0.88471 | 11.4479 | 0.0000 | 0.01979 |
| RFL | -0.00062 | 0.00000 | 0.26236 | -1.31622 | 0.05501 | -9.04847 | 224.247 | 0.0000 | 0.04285 |
| 7011 | 0.00099 | 0.00000 | 2.26603 | -0.09629 | 0.04829 | 40.2957 | 1890.62 | 0.0000 | 0.01777 |
| TXT | 0.00031 | 0.00000 | 0.15015 | -0.18789 | 0.02066 | -0.44608 | 14.5341 | 0.0000 | 0.01884 |
| FCT | -0.00014 | 0.00000 | 0.18898 | -0.17066 | 0.02358 | 0.03938 | 11.3431 | 0.0000 | 0.02334 |
| CEAD | -0.00253 | -0.00330 | 1.18678 | -0.65566 | 0.07938 | 2.05363 | 33.3052 | 0.0000 | 0.06373 |
| 012450 | 0.00032 | 0.00000 | 0.18630 | -0.23775 | 0.02706 | 0.15045 | 10.7255 | 0.0000 | 0.02548 |
| VVX | 0.00032 | 0.00041 | 0.30048 | -0.59564 | 0.03118 | -2.78466 | 75.5932 | 0.0000 | 0.02345 |
| TDG | 0.00088 | 0.00098 | 0.21836 | -0.24813 | 0.02067 | -0.63232 | 27.1947 | 0.0000 | 0.01694 |
| PH | 0.00050 | 0.00024 | 0.16906 | -0.18258 | 0.01903 | -0.47661 | 15.6153 | 0.0000 | 0.01752 |
| STEG | -0.00001 | 0.00000 | 0.08408 | -0.10447 | 0.01195 | -0.26285 | 10.7151 | 0.0000 | 0.01183 |
| OSK | 0.00030 | 0.00000 | 0.19401 | -0.14871 | 0.02135 | 0.01538 | 9.95308 | 0.0000 | 0.02152 |
| J | 0.00028 | 0.00004 | 0.09789 | -0.12901 | 0.01707 | -0.25474 | 9.26955 | 0.0000 | 0.01687 |
| TDY | 0.00061 | 0.00081 | 0.12504 | -0.25990 | 0.01693 | -1.56881 | 30.5172 | 0.0000 | 0.01600 |
| ASELS | 0.00146 | 0.00000 | 0.58854 | -0.49419 | 0.02839 | 1.23521 | 112.135 | 0.0000 | 0.02250 |
| 2302 | 0.00005 | 0.00000 | 0.48327 | -0.27599 | 0.03261 | 2.07528 | 39.5192 | 0.0000 | 0.01262 |
| TKAG | -0.00040 | 0.00000 | 0.24775 | -0.19991 | 0.02824 | -0.03904 | 12.1788 | 0.0000 | 0.02649 |
| BAJE | 0.00111 | 0.00000 | 0.16451 | -0.18173 | 0.02298 | 0.08716 | 8.92526 | 0.0000 | 0.02304 |
| SRP | -0.00036 | 0.00000 | 0.15319 | -0.38857 | 0.02254 | -2.36873 | 48.5908 | 0.0000 | 0.01797 |
| 7012 | 0.00076 | 0.00000 | 2.26711 | -0.11617 | 0.04977 | 36.8690 | 1680.35 | 0.0000 | 0.02186 |
| 079550 | 0.00025 | 0.00000 | 0.26174 | -0.19258 | 0.02725 | 0.51010 | 10.8551 | 0.0000 | 0.02563 |
| BWXT | 0.00045 | 0.00032 | 0.13699 | -0.27117 | 0.01666 | -1.79019 | 37.7578 | 0.0000 | 0.01512 |
| HAGG | 0.00091 | 0.00000 | 0.35465 | -0.20479 | 0.02809 | 2.24265 | 39.0250 | 0.0000 | 0.02573 |
| QQ | 0.00014 | 0.00000 | 0.10597 | -0.14137 | 0.01596 | -0.06570 | 13.0278 | 0.0000 | 0.01488 |
| PGZ | 0.00021 | 0.00000 | 1.38629 | -0.69315 | 0.10077 | 2.22670 | 51.7254 | 0.0000 | 0.00000 |
| 047810 | 0.00021 | 0.00000 | 0.16399 | -0.35382 | 0.02489 | -1.11617 | 23.7531 | 0.0000 | 0.02279 |
| PSN | 0.00062 | 0.00035 | 0.11308 | -0.15994 | 0.02074 | -0.79507 | 12.5949 | 0.0000 | 0.01947 |
| ETN | 0.00045 | 0.00023 | 0.20916 | -0.12747 | 0.01718 | 0.16473 | 17.5388 | 0.0000 | 0.01617 |
| CAE | 0.00029 | 0.00000 | 0.16731 | -0.24026 | 0.02059 | -0.96460 | 30.0755 | 0.0000 | 0.01556 |
| CW | 0.00050 | 0.00041 | 0.12055 | -0.19728 | 0.01791 | -0.57150 | 14.7189 | 0.0000 | 0.01688 |
| MOGa | 0.00030 | 0.00012 | 0.16551 | -0.23383 | 0.02192 | -0.58653 | 17.7088 | 0.0000 | 0.02013 |
| 6755 | 0.00029 | 0.00000 | 0.14427 | -0.13600 | 0.01978 | 0.20506 | 9.49678 | 0.0000 | 0.01954 |
| KOG | 0.00050 | 0.00000 | 0.12097 | -0.20899 | 0.01819 | -0.72645 | 15.5992 | 0.0000 | 0.01692 |
| APH | 0.00058 | 0.00083 | 0.09635 | -0.15252 | 0.01464 | -0.72456 | 12.5690 | 0.0000 | 0.01365 |
| MRON | 0.00060 | 0.00000 | 0.37723 | -0.19369 | 0.02608 | 1.18313 | 27.8699 | 0.0000 | 0.02256 |
| MAZG | 0.00310 | 0.00000 | 0.18028 | -0.12235 | 0.02999 | 1.15121 | 8.14975 | 0.0000 | 0.02689 |
| ASB | 0.00031 | 0.00000 | 0.22813 | -0.29355 | 0.02631 | -0.36965 | 18.7248 | 0.0000 | 0.02340 |
| MRCY | 0.00047 | 0.00000 | 0.17551 | -0.20680 | 0.02499 | -0.19217 | 13.9548 | 0.0000 | 0.02275 |
| BALL | 0.00032 | 0.00027 | 0.12570 | -0.20558 | 0.01711 | -0.31409 | 17.5808 | 0.0000 | 0.01582 |
| HWM | 0.00078 | 0.00095 | 0.23375 | -0.23117 | 0.02703 | -0.37756 | 17.0974 | 0.0000 | 0.02221 |
| TTMI | 0.00025 | 0.00000 | 0.20424 | -0.20501 | 0.02485 | -0.07247 | 15.5024 | 0.0000 | 0.02057 |
| HEI | 0.00070 | 0.00068 | 0.11831 | -0.17887 | 0.01873 | -0.17056 | 11.3762 | 0.0000 | 0.01757 |
| 064350 | -0.00004 | 0.00000 | 0.26236 | -0.21309 | 0.02919 | 0.84043 | 15.8704 | 0.0000 | 0.02513 |
| IHI | 0.00070 | 0.00000 | 2.28832 | -0.22619 | 0.05172 | 33.7247 | 1495.43 | 0.0000 | 0.02406 |

Note: Descriptive statistics include mean, median, maximum, minimum, standard deviation for variability, skewness and kurtosis for distribution shape, and IQR (Inter-Quartile Range) as a measure of spread less sensitive to outliers. The probability value provides the p-value for the normality test. Source: Author’s computation based on historical daily returns.
