# Supplementary material for: Investigating the effect of geopolitical risk on defense companies’ stock returns
Source: Heliyon. 2024 Dec 7;10(24):e40974. doi: 10.1016/j.heliyon.2024.e40974 (PMC11700249; doi:10.1016/j.heliyon.2024.e40974)
Supplement: Multimedia component 2 [file mmc2.docx]

Appendix 2

The graphic trends of each time series

|  |  |  |
| --- | --- | --- |
|  |  |  |
|  |  |  |
|  |  |  |
|  |  |  |
|  |  |  |
|  |  |  |
|  |  |  |
|  |  |  |
|  |  |  |
|  |  |  |
|  |  |  |
|  |  |  |
|  |  |  |
|  |  |  |
|  |  |  |
|  |  |  |
|  |  |  |
|  |  |  |
|  |  |  |
|  |  |  |
|  |  |  |
|  |  |  |
|  |  |  |
|  |  |  |

Note: Appendix 2 illustrates the graphic trends for each time series, providing a visual overview of fluctuations in defense stock returns over the analyzed period. Source: Author’s computation based on historical daily returns.
