# Supplementary material for: Investigating the effect of geopolitical risk on defense companies’ stock returns
Source: Heliyon. 2024 Dec 7;10(24):e40974. doi: 10.1016/j.heliyon.2024.e40974 (PMC11700249; doi:10.1016/j.heliyon.2024.e40974)
Supplement: Multimedia component 3 [file mmc3.docx]

Appendix 3

Scalogram of stocks

| 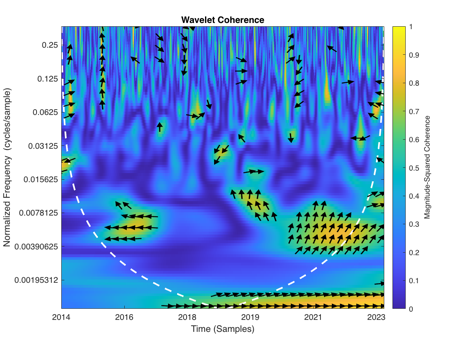 | 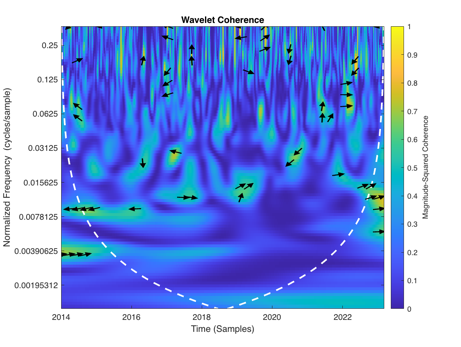 | 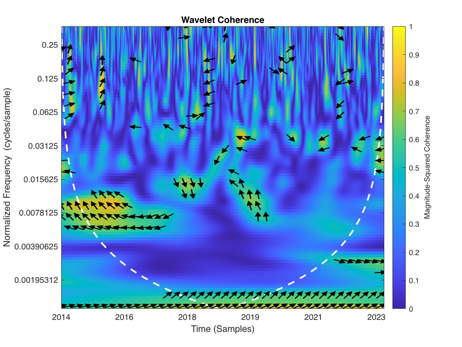 |
| --- | --- | --- |
| LMT | RYTT34 | NOC |
| 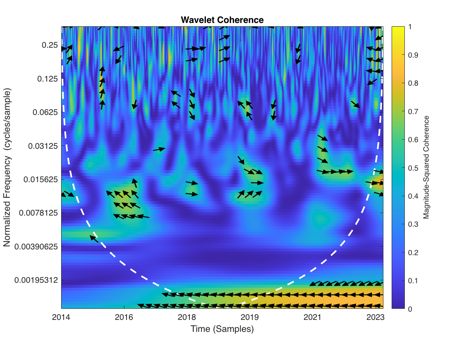 | 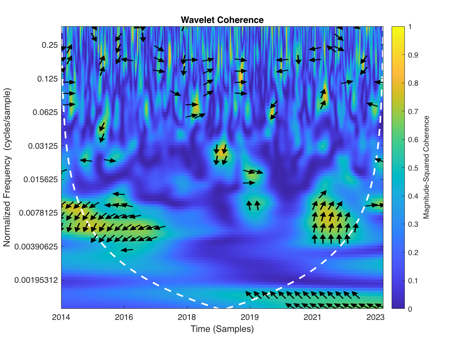 | 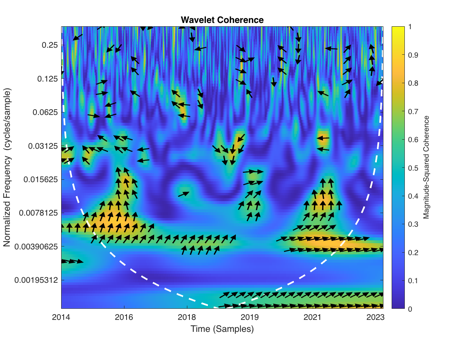 |
| BA | GD | BAES |
| 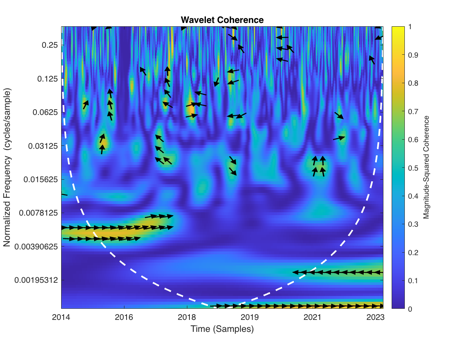 | 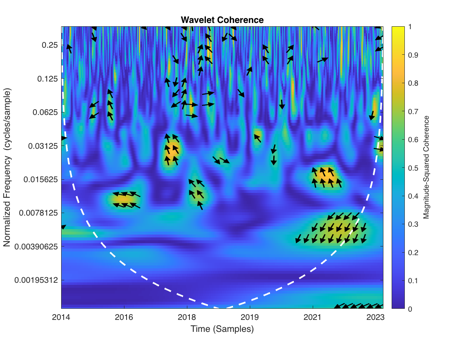 | 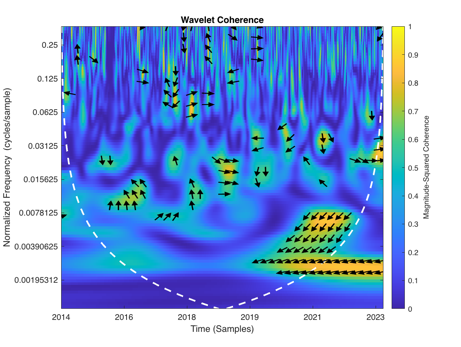 |
| 000065 | 000768 | 600879 |
| 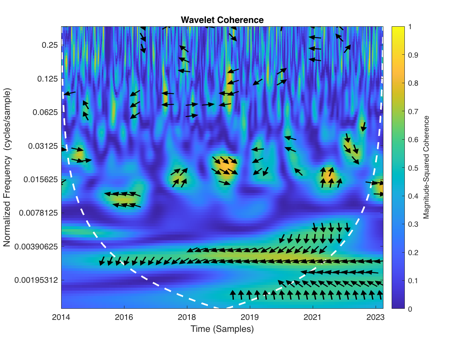 | 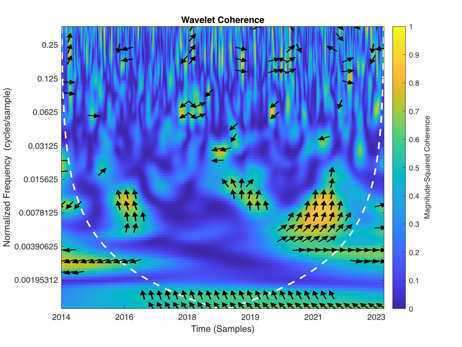 | 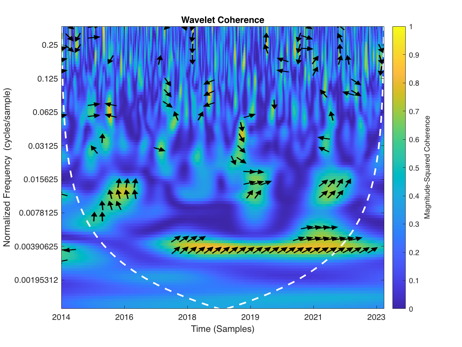 |
| 002268 | LHX | LDOF |
| 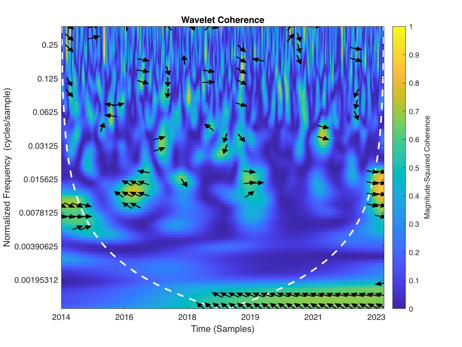 | 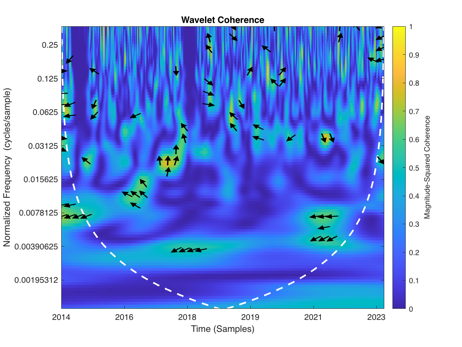 | 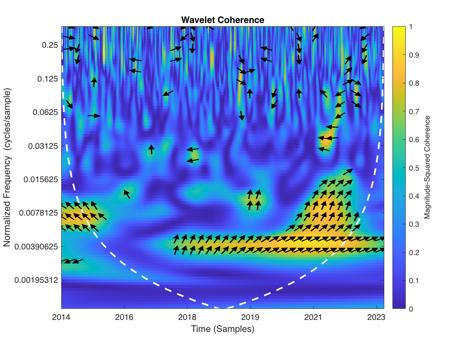 |
| AIR | 600685 | TCFP |
| 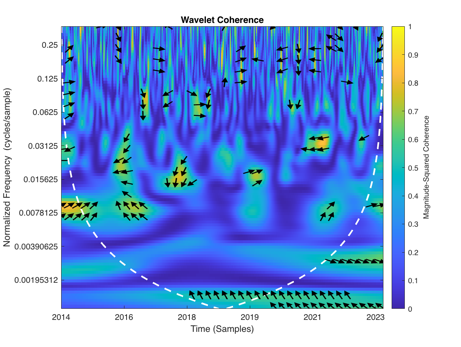 | 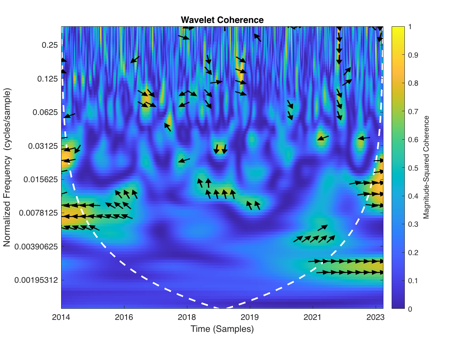 | 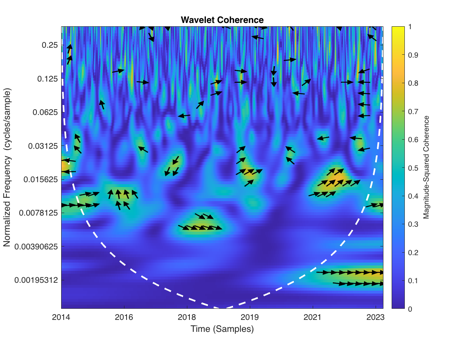 |
| HII | LDOS | BAH |
| 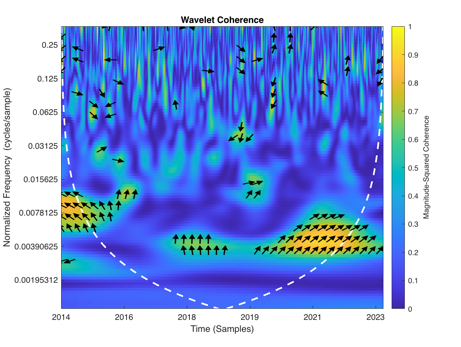 | 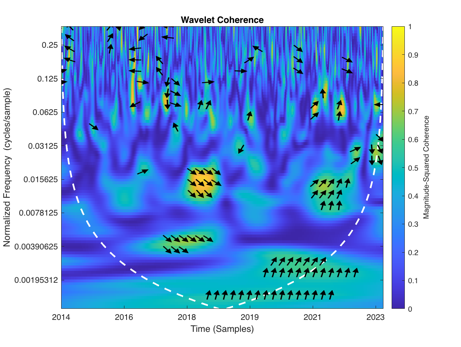 | 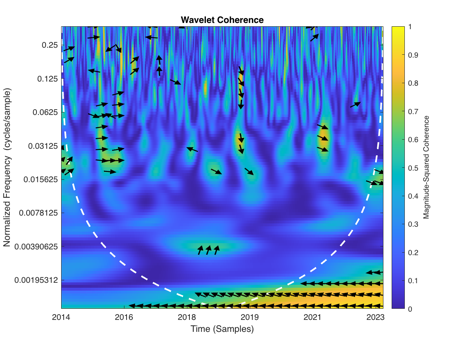 |
| AM | ESLT | RR |
| 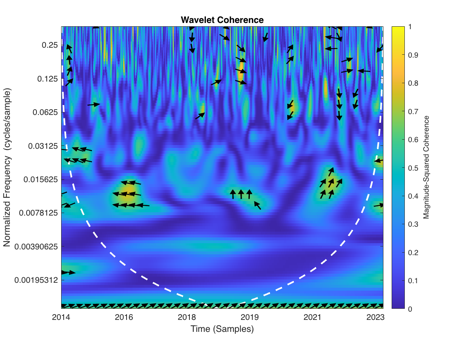 | 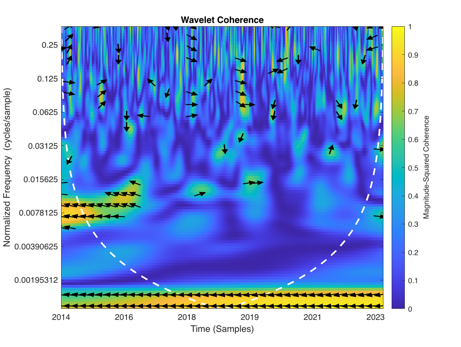 | 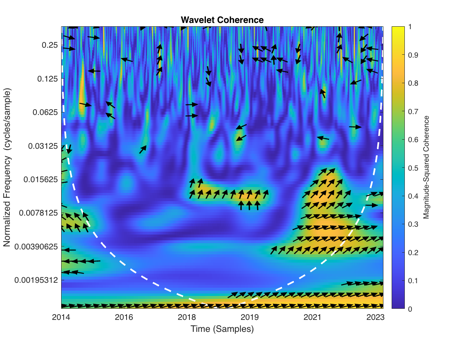 |
| CACI | HON | RHMG |
| 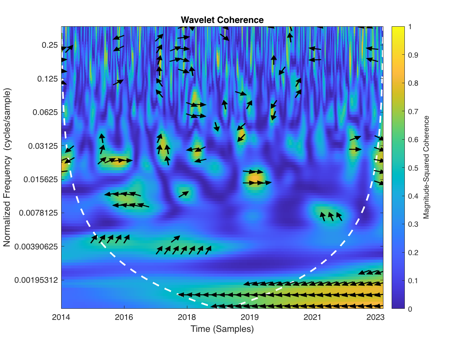 | 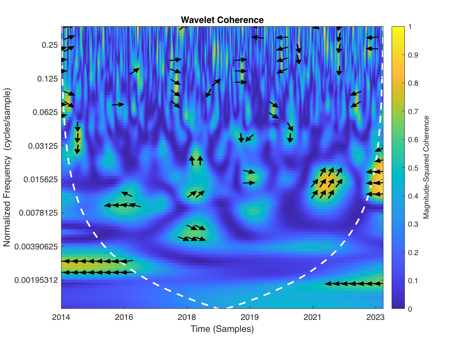 | 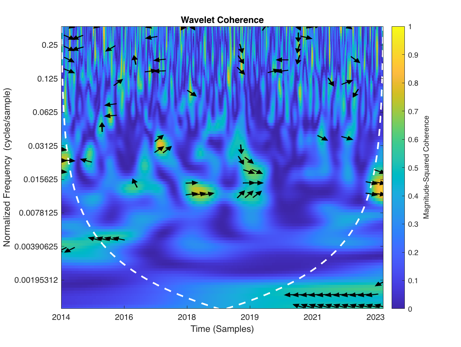 |
| GE | KBR | SAF |
| 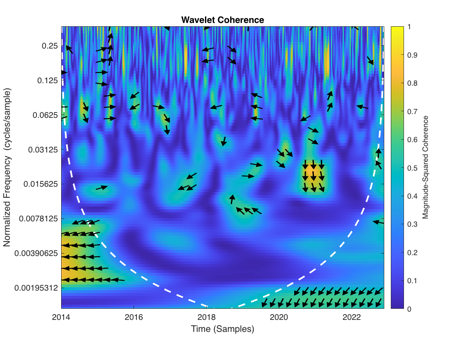 | 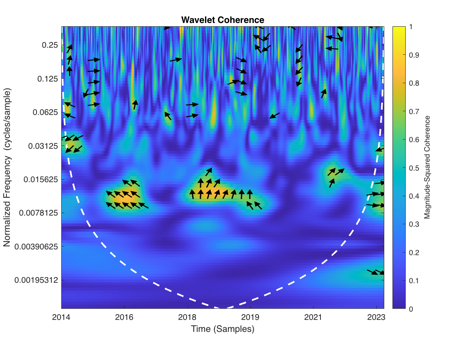 | 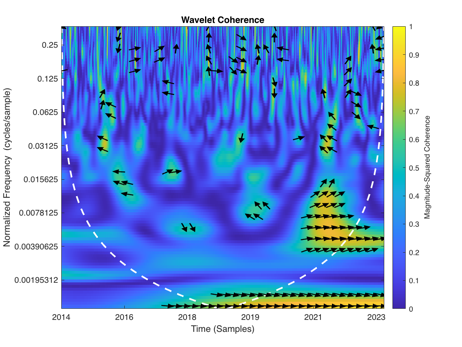 |
| ILARSP4=TA | SAIC | SAABBs |
| 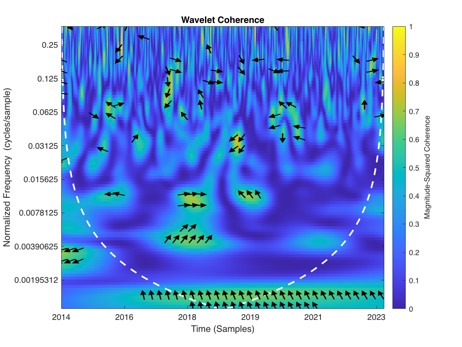 | 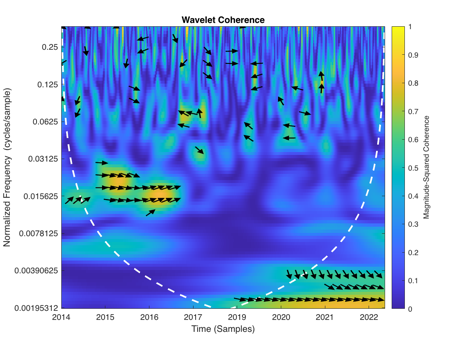 | 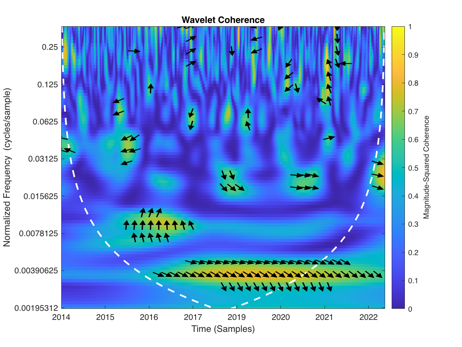 |
| BAB | HIAE | RFL |
| 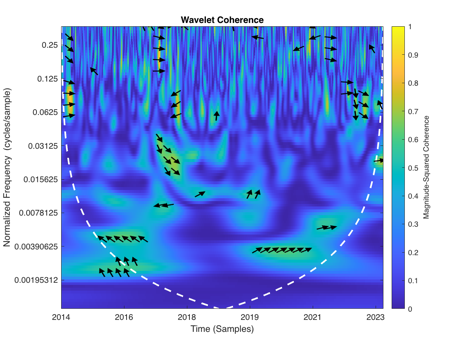 | 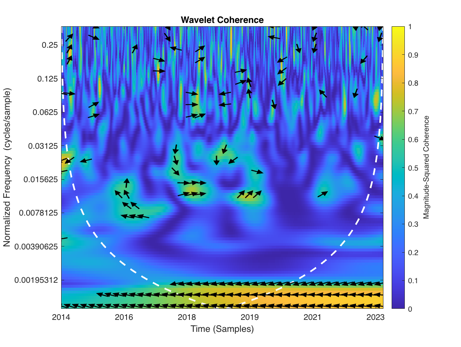 | 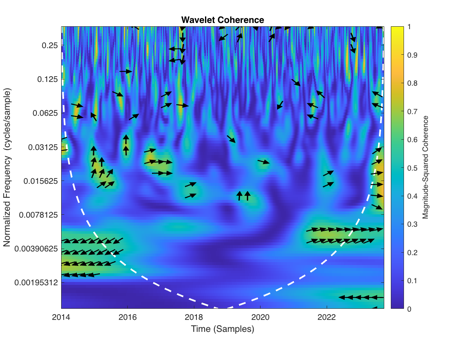 |
| 7011 | TXT | FCT |
| 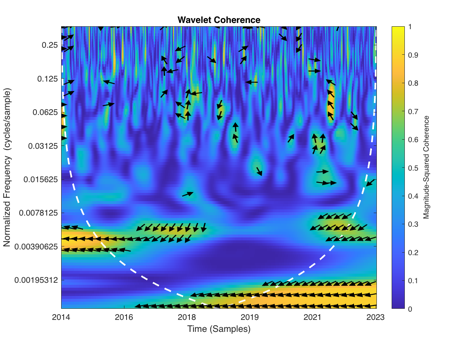 | 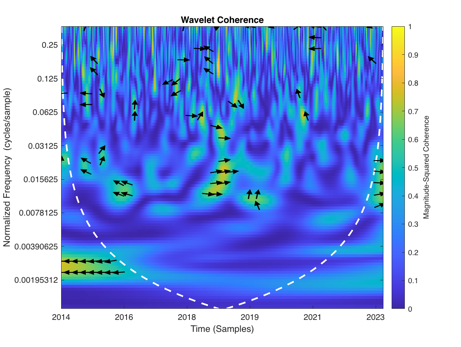 | 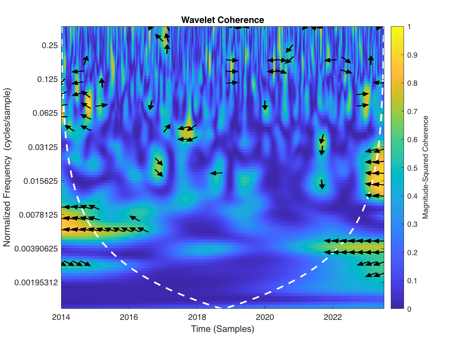 |
| CEAD | 012450 | VVX |
| 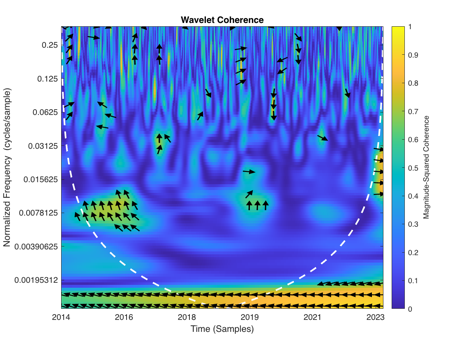 | 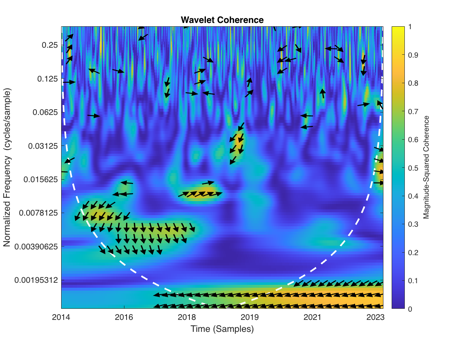 | 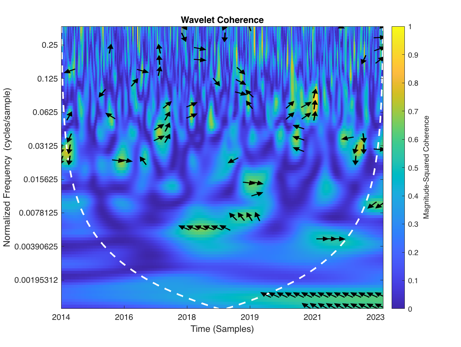 |
| TDG | PH | STEG |
| 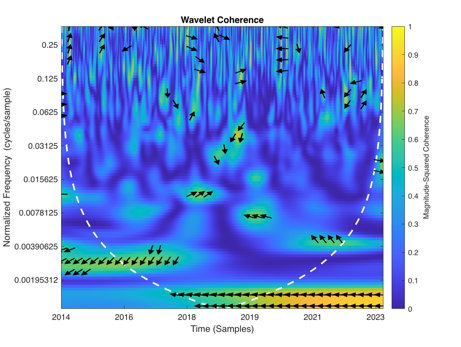 | 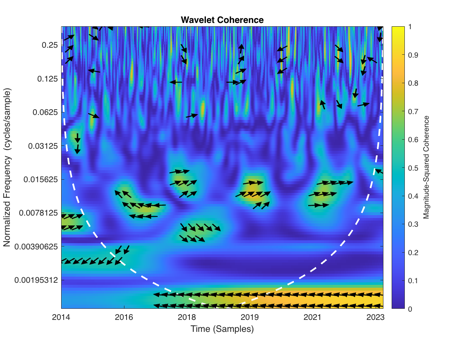 | 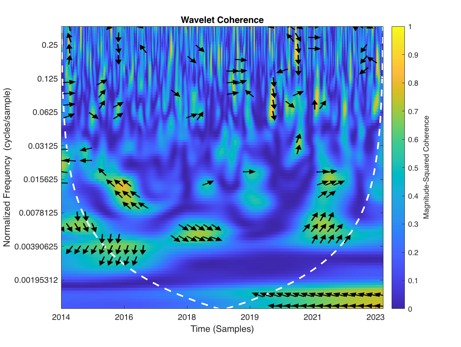 |
| OSK | J | TDY |
| 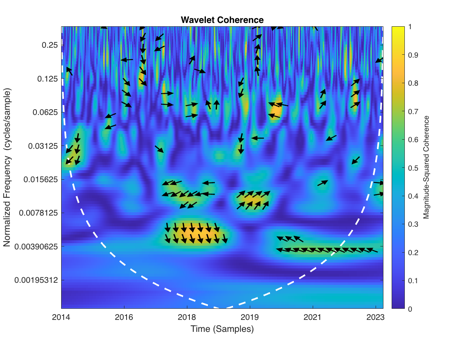 | 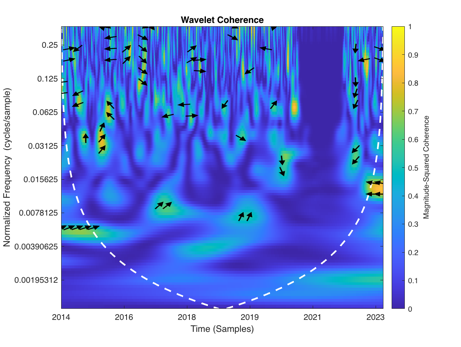 | 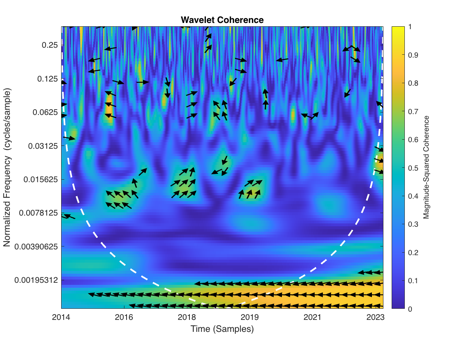 |
| ASELS | 2302 | TKAG |
| 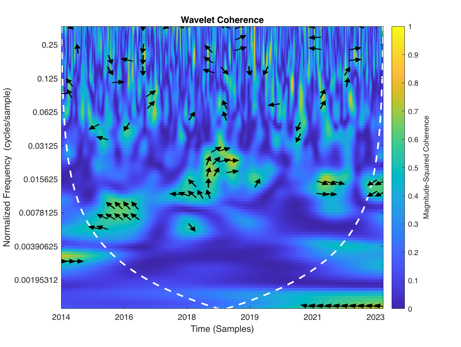 | 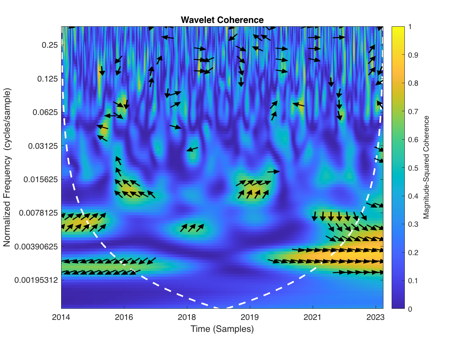 | 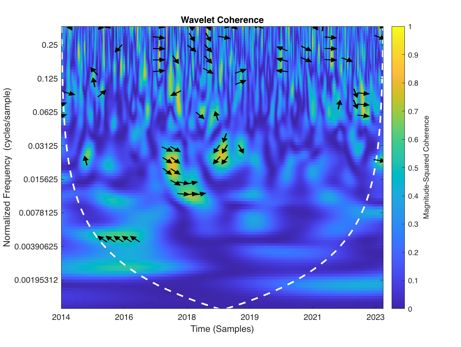 |
| BAJE | SRP | 7012 |
| 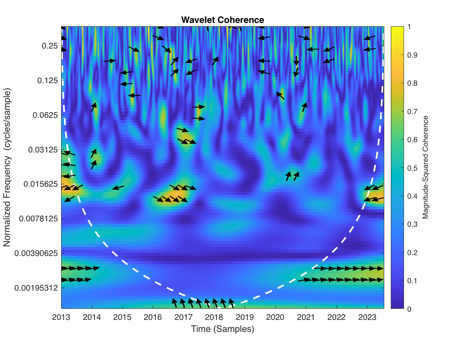 | 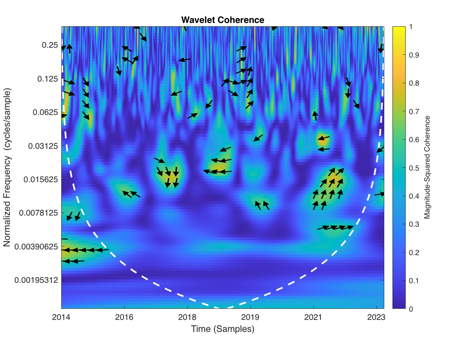 | 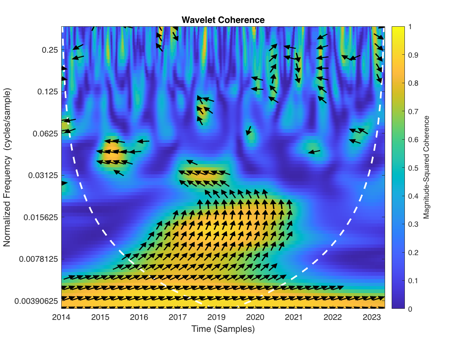 |
| 079550 | BWXT | HAGG |
| 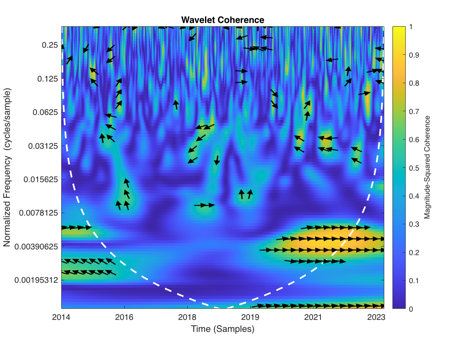 | 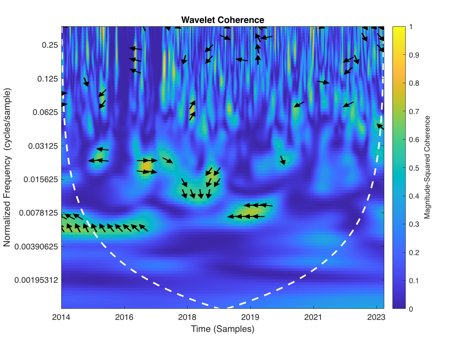 | 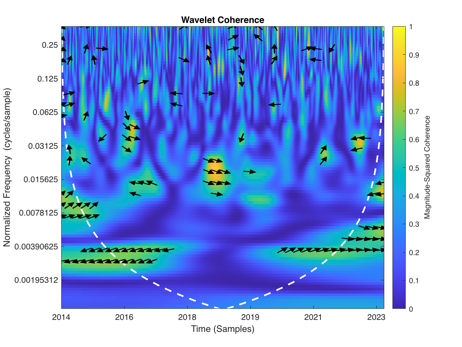 |
| QQ | PGZ | 047810 |
| 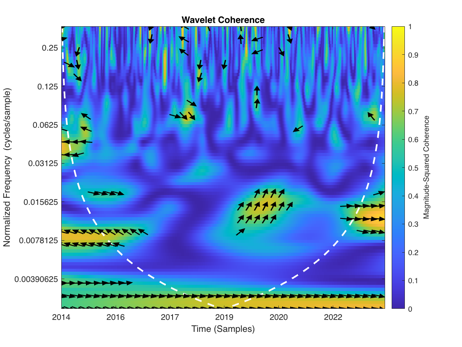 | 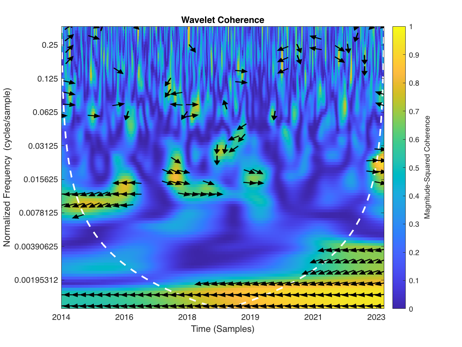 | 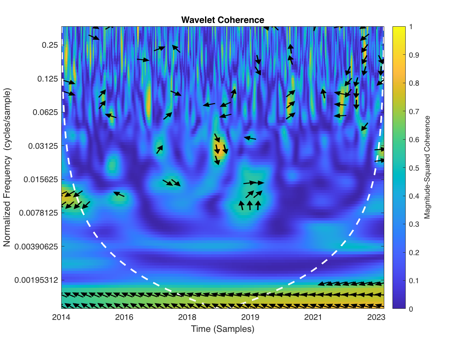 |
| PSN | ETN | CAE |
| 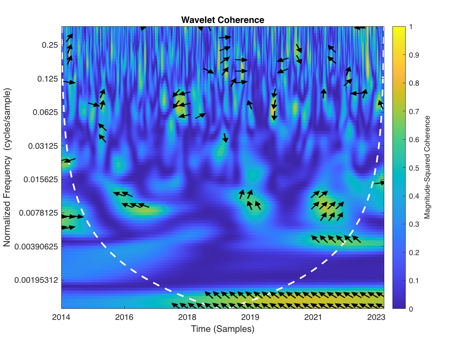 | 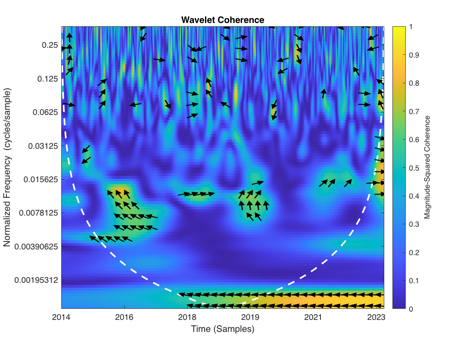 | 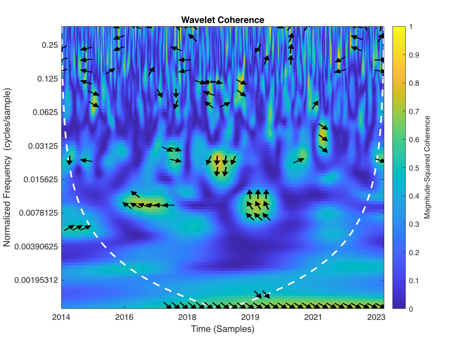 |
| CW | MOGa | 6755 |
| 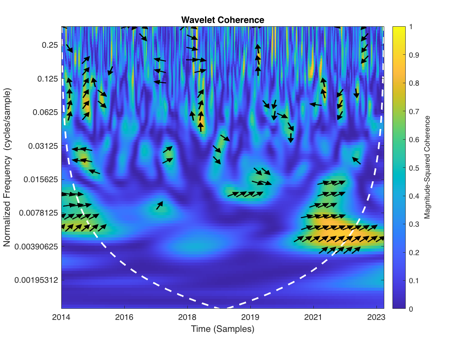 | 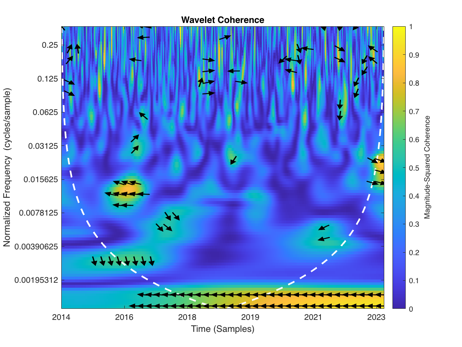 | 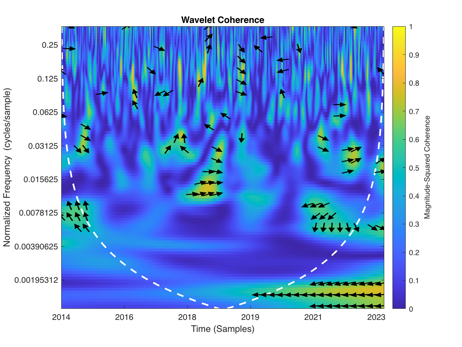 |
| KOG | APH | MRON |
| 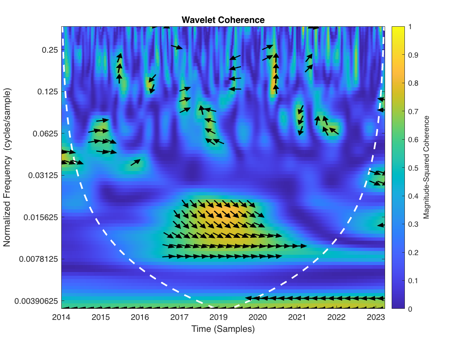 | 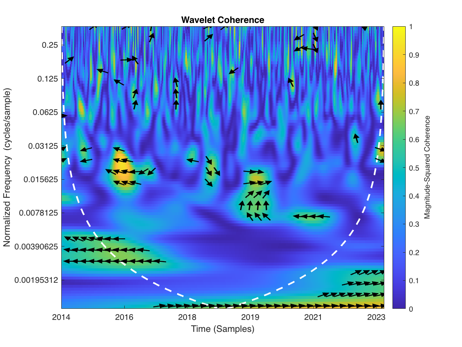 | 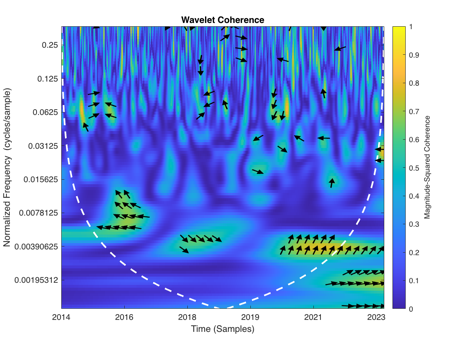 |
| MAZG | ASB | MRCY |
| 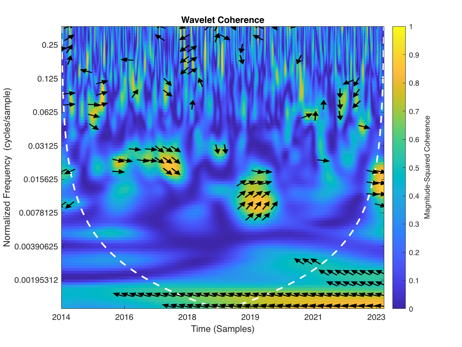 | 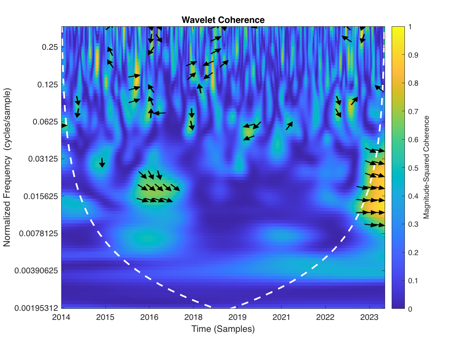 | 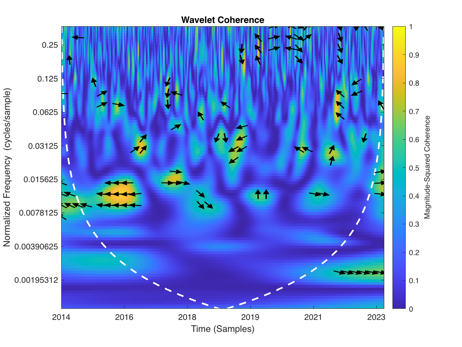 |
| BALL | HWM | TTMI |
|  |  |  |
| 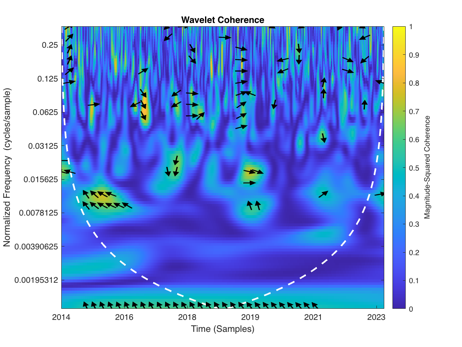 | 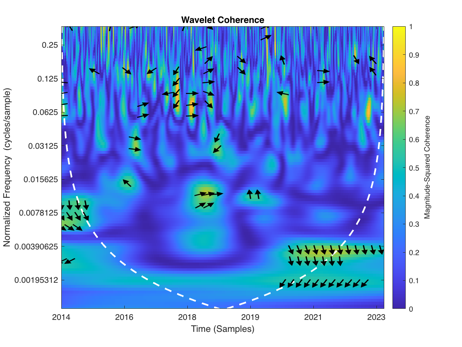 | 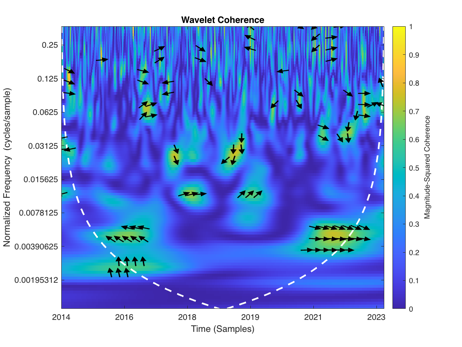 |
| HEI | 064350 | 7013 |

Note: Appendix 3 provides scalograms illustrating the time-frequency dynamics of defense stock returns, highlighting periods of significant coherence with the GPR index. Source: Author’s computation based on historical daily returns.
