# Supplementary material for: Investigating the effect of geopolitical risk on defense companies’ stock returns
Source: Heliyon. 2024 Dec 7;10(24):e40974. doi: 10.1016/j.heliyon.2024.e40974 (PMC11700249; doi:10.1016/j.heliyon.2024.e40974)
Supplement: Multimedia component 4 [file mmc4.docx]

Appendix 4

The outcomes of unit root examinations

| ADF | t-Statistic | Prob.* | ADF | t-Statistic | Prob.* | ADF | t-Statistic | Prob.* |
| --- | --- | --- | --- | --- | --- | --- | --- | --- |
| LMT | -51.44006 | 0.0001 | RYTT34 | -16.9792 | 0.0000 | NOC | -53.43632 | 0.0001 |
| 1% level | -3.43271 |  | 1% level | -3.433015 |  | 1% level | -3.43271 |  |
| 5% level | -2.862469 |  | 5% level | -2.862603 |  | 5% level | -2.862469 |  |
| 10% level | -2.567309 |  | 10% level | -2.567382 |  | 10% level | -2.567309 |  |
| BA | -19.38009 | 0.0000 | GD | -51.1686 | 0.0001 | BAES | -49.05572 | 0.0001 |
| 1% level | -3.432716 |  | 1% level | -3.43271 |  | 1% level | -3.43271 |  |
| 5% level | -2.862471 |  | 5% level | -2.862469 |  | 5% level | -2.862469 |  |
| 10% level | -2.567311 |  | 10% level | -2.567309 |  | 10% level | -2.567309 |  |
| 000065 | -44.30853 | 0.0001 | 000768 | -46.70475 | 0.0001 | 600879 | -49.54913 | 0.0001 |
| 1% level | -3.43271 |  | 1% level | -3.43271 |  | 1% level | -3.43271 |  |
| 5% level | -2.862469 |  | 5% level | -2.862469 |  | 5% level | -2.862469 |  |
| 10% level | -2.567309 |  | 10% level | -2.567309 |  | 10% level | -2.567309 |  |
| 002268 | -47.75224 | 0.0001 | LHX | -53.06176 | 0.0001 | LDOF | -49.04300 | 0.0001 |
| 1% level | -3.43271 |  | 1% level | -3.43271 |  | 1% level | -3.43271 |  |
| 5% level | -2.862469 |  | 5% level | -2.862469 |  | 5% level | -2.862469 |  |
| 10% level | -2.567309 |  | 10% level | -2.567309 |  | 10% level | -2.567309 |  |
| AIR | -19.33313 | 0.0000 | 600685 | -45.63525 | 0.0001 | TCFP | -48.49354 | 0.0001 |
| 1% level | -3.432716 |  | 1% level | -3.43271 |  | 1% level | -3.43271 |  |
| 5% level | -2.862471 |  | 5% level | -2.862469 |  | 5% level | -2.862469 |  |
| 10% level | -2.567311 |  | 10% level | -2.567309 |  | 10% level | -2.567309 |  |
| HII | -51.19464 | 0.0001 | LDOS | -51.34285 | 0.0001 | BAH | -51.64773 | 0.0001 |
| 1% level | -3.43271 |  | 1% level | -3.43271 |  | 1% level | -3.43271 |  |
| 5% level | -2.862469 |  | 5% level | -2.862469 |  | 5% level | -2.862469 |  |
| 10% level | -2.567309 |  | 10% level | -2.567309 |  | 10% level | -2.567309 |  |
| AM | -50.81138 | 0.0001 | ESLT | -54.13857 | 0.0001 | RR | -49.18164 | 0.0001 |
| 1% level | -3.43271 |  | 1% level | -3.43271 |  | 1% level | -3.43271 |  |
| 5% level | -2.862469 |  | 5% level | -2.862469 |  | 5% level | -2.862469 |  |
| 10% level | -2.567309 |  | 10% level | -2.567309 |  | 10% level | -2.567309 |  |
| CACI | -34.29494 | 0.0000 | HON | -19.31336 | 0.0000 | RHMG | -46.21486 | 0.0001 |
| 1% level | -3.432711 |  | 1% level | -3.432716 |  | 1% level | -3.43271 |  |
| 5% level | -2.862469 |  | 5% level | -2.862471 |  | 5% level | -2.862469 |  |
| 10% level | -2.56731 |  | 10% level | -2.567311 |  | 10% level | -2.567309 |  |
| GE | -51.20403 | 0.0001 | KBR | -33.95965 | 0.0000 | SAF | -24.81649 | 0.0000 |
| 1% level | -3.43271 |  | 1% level | -3.432711 |  | 1% level | -3.432715 |  |
| 5% level | -2.862469 |  | 5% level | -2.862469 |  | 5% level | -2.862471 |  |
| 10% level | -2.567309 |  | 10% level | -2.56731 |  | 10% level | -2.56731 |  |
| ILARSP4=TA | -37.60183 | 0.0000 | SAIC | -34.81707 | 0.0000 | SAABBs | -48.55568 | 0.0001 |
| 1% level | -3.433096 |  | 1% level | -3.432711 |  | 1% level | -3.43271 |  |
| 5% level | -2.862639 |  | 5% level | -2.862469 |  | 5% level | -2.862469 |  |
| 10% level | -2.567401 |  | 10% level | -2.56731 |  | 10% level | -2.567309 |  |
| BAB | -48.61274 | 0.0001 | HIAE | -35.51482 | 0.0000 | RFL | -38.03687 | 0.0000 |
| 1% level | -3.43271 |  | 1% level | -3.434582 |  | 1% level | -3.434576 |  |
| 5% level | -2.862469 |  | 5% level | -2.863296 |  | 5% level | -2.863293 |  |
| 10% level | -2.567309 |  | 10% level | -2.567753 |  | 10% level | -2.567752 |  |
| 7011 | -51.07305 | 0.0001 | TXT | -17.78481 | 0.0000 | FCT | -45.48437 | 0.0001 |
| 1% level | -3.43271 |  | 1% level | -3.432716 |  | 1% level | -3.432844 |  |
| 5% level | -2.862469 |  | 5% level | -2.862471 |  | 5% level | -2.862528 |  |
| 10% level | -2.567309 |  | 10% level | -2.567311 |  | 10% level | -2.567341 |  |
| CEAD | -51.46466 | 0.0001 | 012450 | -33.52998 | 0.0000 | VVX | -53.01172 | 0.0001 |
| 1% level | -3.432763 |  | 1% level | -3.432711 |  | 1% level | -3.432905 |  |
| 5% level | -2.862492 |  | 5% level | -2.862469 |  | 5% level | -2.862555 |  |
| 10% level | -2.567322 |  | 10% level | -2.56731 |  | 10% level | -2.567356 |  |
| TDG | -20.16614 | 0.0000 | PH | -18.45929 | 0.0000 | STEG | -54.35343 | 0.0001 |
| 1% level | -3.432716 |  | 1% level | -3.432716 |  | 1% level | -3.43271 |  |
| 5% level | -2.862471 |  | 5% level | -2.862471 |  | 5% level | -2.862469 |  |
| 10% level | -2.567311 |  | 10% level | -2.567311 |  | 10% level | -2.567309 |  |
| OSK | -51.2011 | 0.0001 | J | -50.77191 | 0.0001 | TDY | -18.8874 | 0.0000 |
| 1% level | -3.43271 |  | 1% level | -3.43271 |  | 1% level | -3.432716 |  |
| 5% level | -2.862469 |  | 5% level | -2.862469 |  | 5% level | -2.862471 |  |
| 10% level | -2.567309 |  | 10% level | -2.567309 |  | 10% level | -2.567311 |  |
| ASELS | -24.95253 | 0.0000 | 2302 | -32.29687 | 0.0000 | TKAG | -48.16306 | 0.0001 |
| 1% level | -3.432714 |  | 1% level | -3.432711 |  | 1% level | -3.43271 |  |
| 5% level | -2.86247 |  | 5% level | -2.862469 |  | 5% level | -2.862469 |  |
| 10% level | -2.56731 |  | 10% level | -2.56731 |  | 10% level | -2.567309 |  |
| BAJE | -50.97606 | 0.0001 | SRP | -49.16432 | 0.0001 | 7012 | -50.93944 | 0.0001 |
| 1% level | -3.43271 |  | 1% level | -3.43271 |  | 1% level | -3.43271 |  |
| 5% level | -2.862469 |  | 5% level | -2.862469 |  | 5% level | -2.862469 |  |
| 10% level | -2.567309 |  | 10% level | -2.567309 |  | 10% level | -2.567309 |  |
| 079550 | -45.66572 | 0.0001 | BWXT | -50.91726 | 0.0001 | HAGG | -24.37292 | 0.0000 |
| 1% level | -3.43325 |  | 1% level | -3.43271 |  | 1% level | -3.437966 |  |
| 5% level | -2.862707 |  | 5% level | -2.862469 |  | 5% level | -2.864791 |  |
| 10% level | -2.567437 |  | 10% level | -2.567309 |  | 10% level | -2.568555 |  |
| QQ | -52.33729 | 0.0001 | PGZ | -25.94923 | 0.0000 | 047810 | -51.72627 | 0.0001 |
| 1% level | -3.43271 |  | 1% level | -3.432715 |  | 1% level | -3.43271 |  |
| 5% level | -2.862469 |  | 5% level | -2.862471 |  | 5% level | -2.862469 |  |
| 10% level | -2.567309 |  | 10% level | -2.56731 |  | 10% level | -2.567309 |  |
| PSN | -22.50839 | 0.0000 | ETN | -18.79956 | 0.0000 | CAE | -18.54263 | 0.0000 |
| 1% level | -3.435631 |  | 1% level | -3.432716 |  | 1% level | -3.432716 |  |
| 5% level | -2.86376 |  | 5% level | -2.862471 |  | 5% level | -2.862471 |  |
| 10% level | -2.568002 |  | 10% level | -2.567311 |  | 10% level | -2.567311 |  |
| CW | -51.99289 | 0.0001 | MOGa | -33.41201 | 0.0000 | 6755 | -49.12809 | 0.0001 |
| 1% level | -3.43271 |  | 1% level | -3.432711 |  | 1% level | -3.43271 |  |
| 5% level | -2.862469 |  | 5% level | -2.862469 |  | 5% level | -2.862469 |  |
| 10% level | -2.567309 |  | 10% level | -2.56731 |  | 10% level | -2.567309 |  |
| KOG | -53.42953 | 0.0001 | APH | -53.16253 | 0.0001 | MRON | -51.07508 | 0.0001 |
| 1% level | -3.43271 |  | 1% level | -3.43271 |  | 1% level | -3.43271 |  |
| 5% level | -2.862469 |  | 5% level | -2.862469 |  | 5% level | -2.862469 |  |
| 10% level | -2.567309 |  | 10% level | -2.567309 |  | 10% level | -2.567309 |  |
| MAZG | -28.15053 | 0.0000 | ASB | -52.32853 | 0.0001 | MRCY | -52.36396 | 0.0001 |
| 1% level | -3.438071 |  | 1% level | -3.43271 |  | 1% level | -3.43271 |  |
| 5% level | -2.864838 |  | 5% level | -2.862469 |  | 5% level | -2.862469 |  |
| 10% level | -2.56858 |  | 10% level | -2.567309 |  | 10% level | -2.567309 |  |
| BALL | -50.85781 | 0.0001 | HWM | -38.95803 | 0.0000 | TTMI | -54.39763 | 0.0001 |
| 1% level | -3.43271 |  | 1% level | -3.434591 |  | 1% level | -3.43271 |  |
| 5% level | -2.862469 |  | 5% level | -2.8633 |  | 5% level | -2.862469 |  |
| 10% level | -2.567309 |  | 10% level | -2.567756 |  | 10% level | -2.567309 |  |
| HEI | -19.57669 | 0.0000 | 064350 | -35.04889 | 0.0000 | 7013 | -50.40545 | 0.0001 |
| 1% level | -3.432716 |  | 1% level | -3.432711 |  | 1% level | -3.43271 |  |
| 5% level | -2.862471 |  | 5% level | -2.862469 |  | 5% level | -2.862469 |  |
| 10% level | -2.567311 |  | 10% level | -2.56731 |  | 10% level | -2.567309 |  |

Note: Appendix 4 present the outcomes of unit root tests using the Augmented Dickey-Fuller (ADF) method. The t-statistic and corresponding p-values assess stationarity in each series, with critical values at the 1%, 5%, and 10% significance levels. Source: Author’s computation based on historical daily returns.
