# Supplementary material for: Investigating the effect of geopolitical risk on defense companies’ stock returns
Source: Heliyon. 2024 Dec 7;10(24):e40974. doi: 10.1016/j.heliyon.2024.e40974 (PMC11700249; doi:10.1016/j.heliyon.2024.e40974)
Supplement: Multimedia component 5 [file mmc5.docx]

Appendix 5

Cross-Wavelet Transform (XWT) between GPR and Stock Returns

| 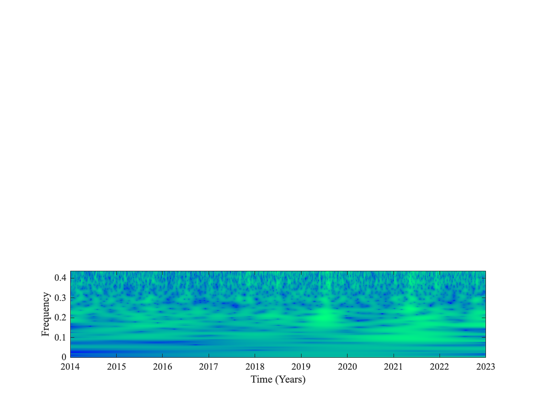 | 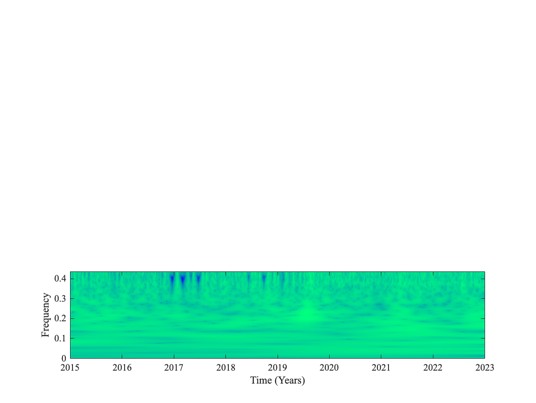 | 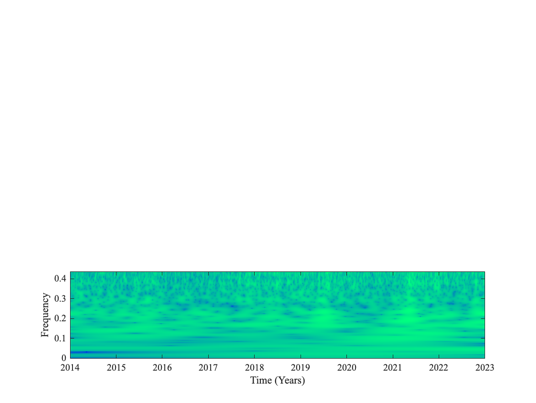 |
| --- | --- | --- |
| LMT | RYTT34 | NOC |
| 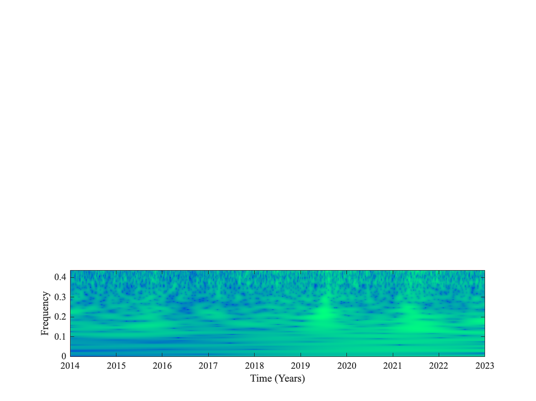 | 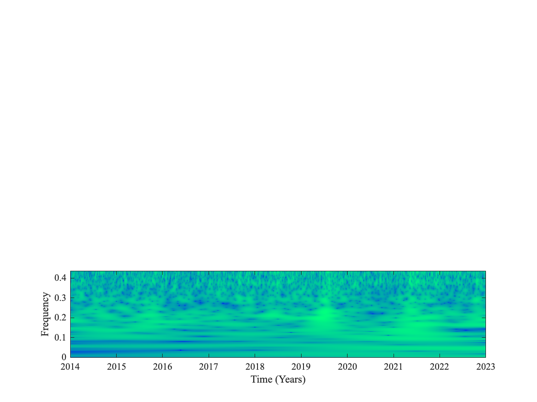 | 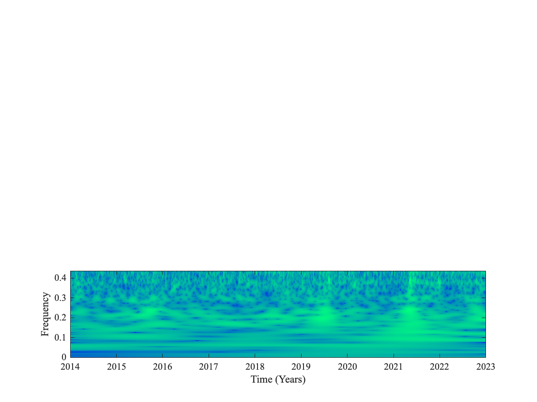 |
| BA | GD | BAES |
| 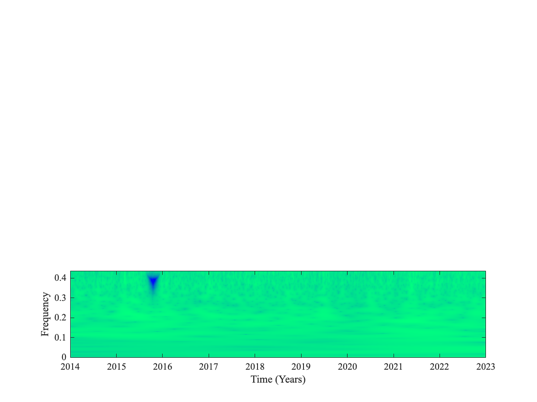 | 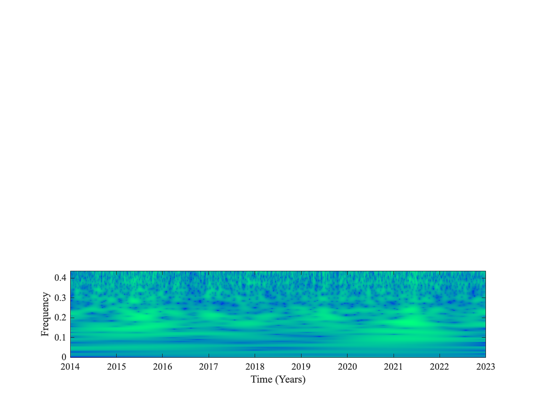 | 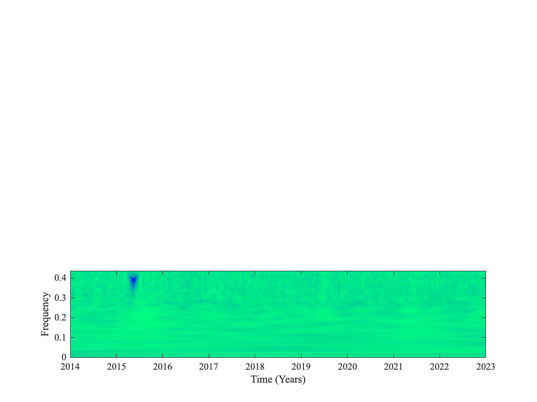 |
| 000065 | 000768 | 600879 |
| 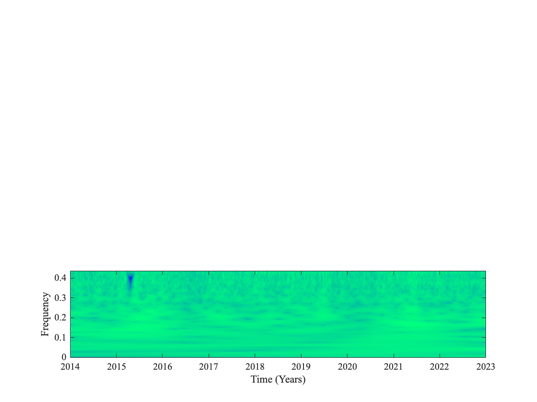 | 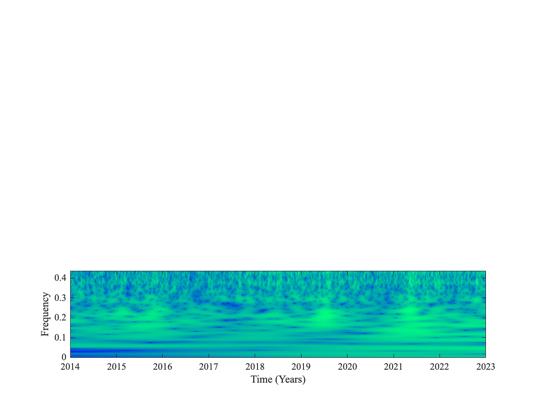 | 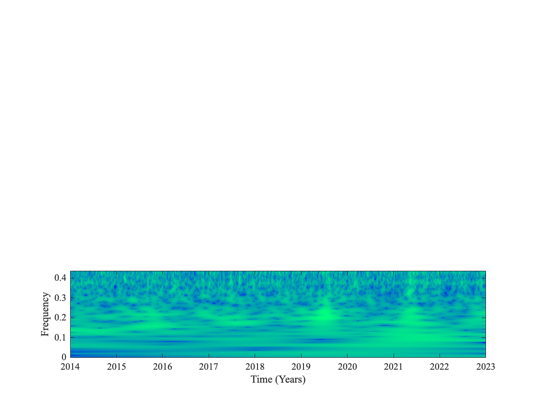 |
| 002268 | LHX | LDOF |
| 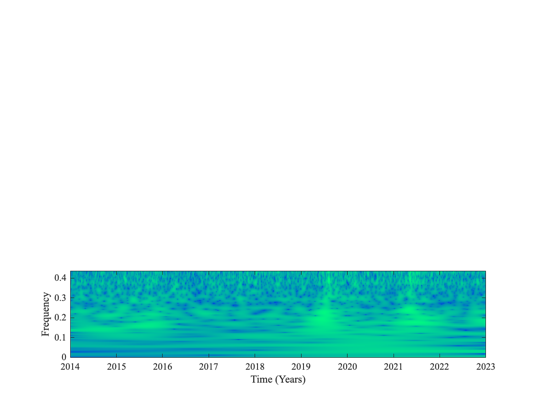 | 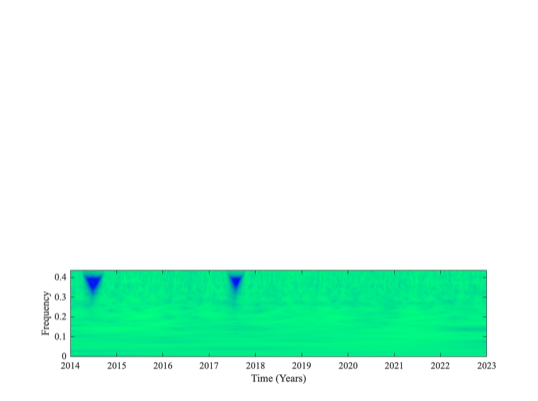 | 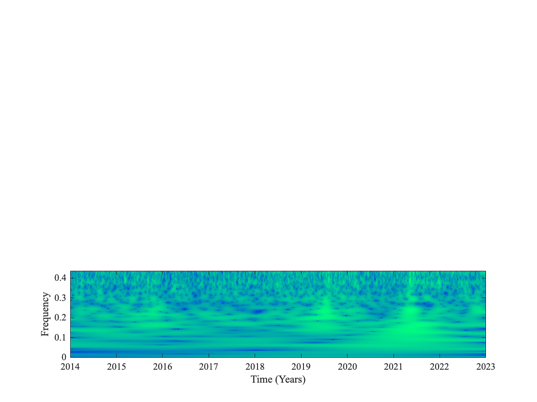 |
| AIR | 600685 | TCFP |
| 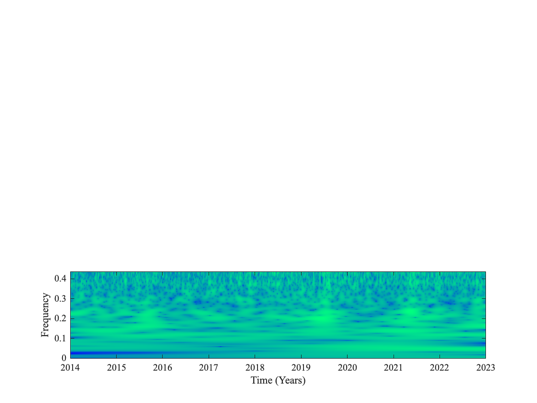 | 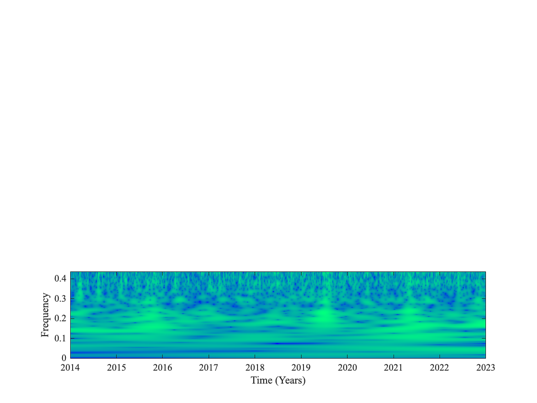 | 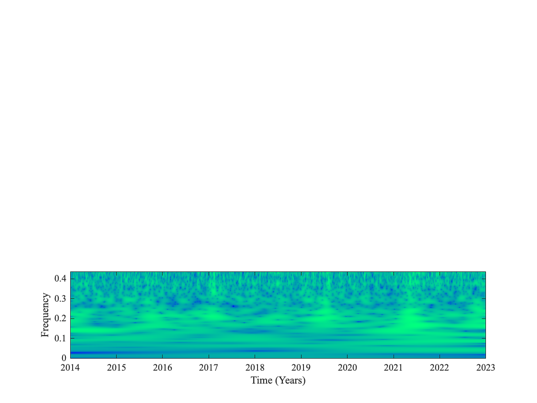 |
| HII | LDOS | BAH |
| 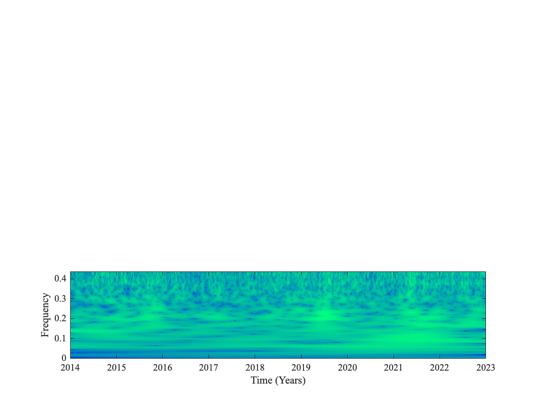 | 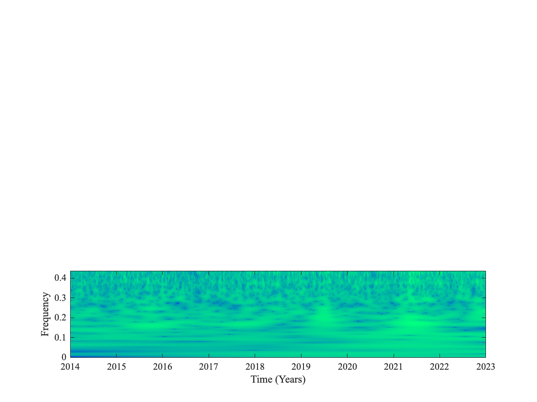 | 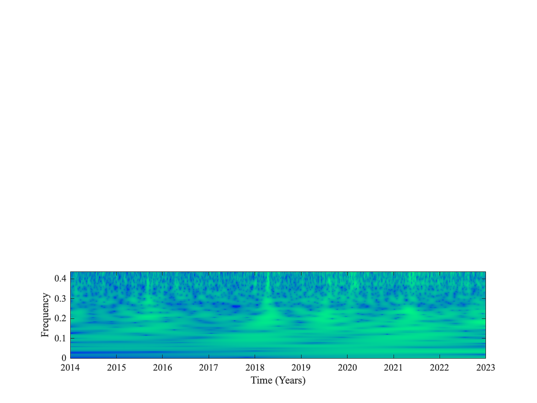 |
| AM | ESLT | RR |
| 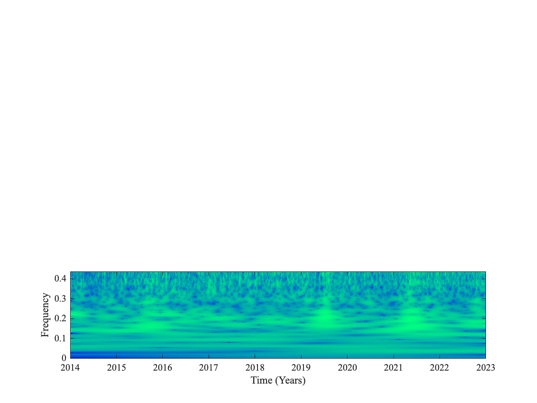 | 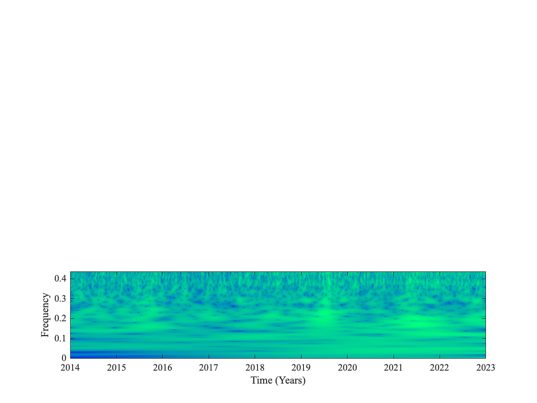 | 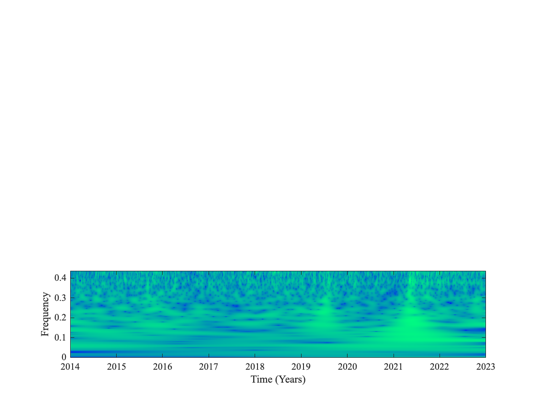 |
| CACI | HON | RHMG |
| 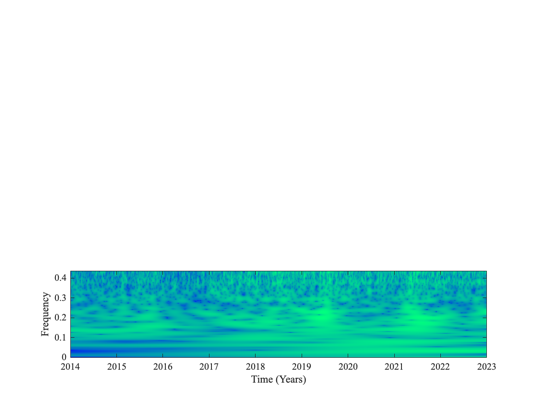 | 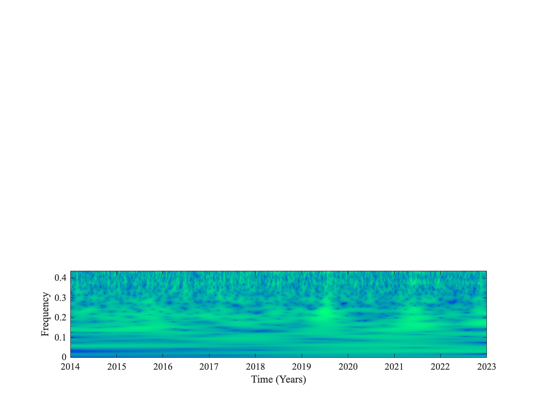 | 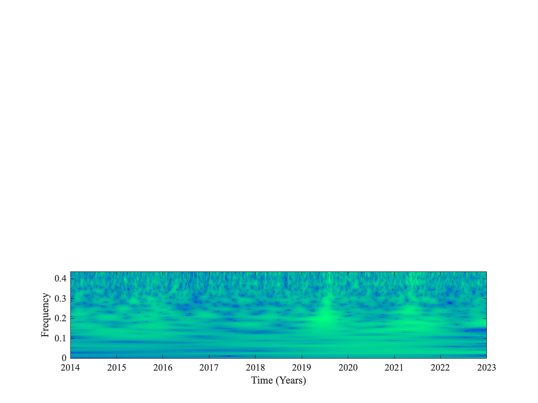 |
| GE | KBR | SAF |
| 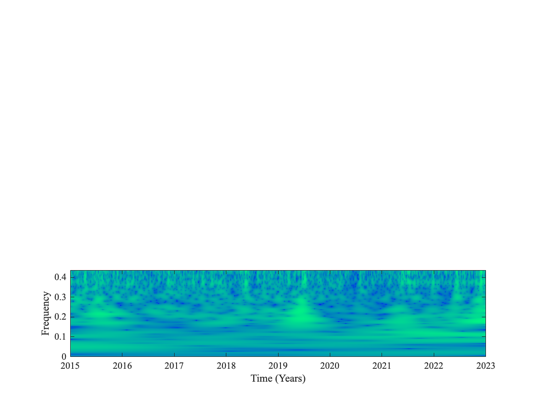 | 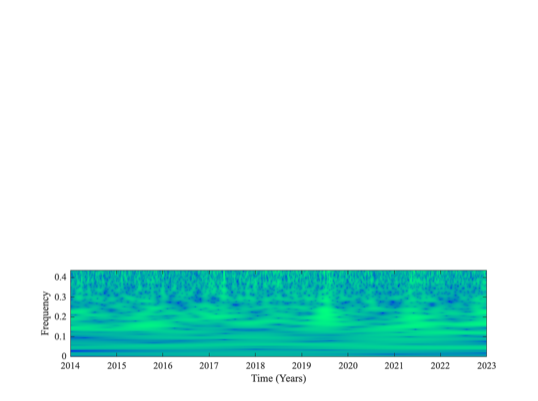 | 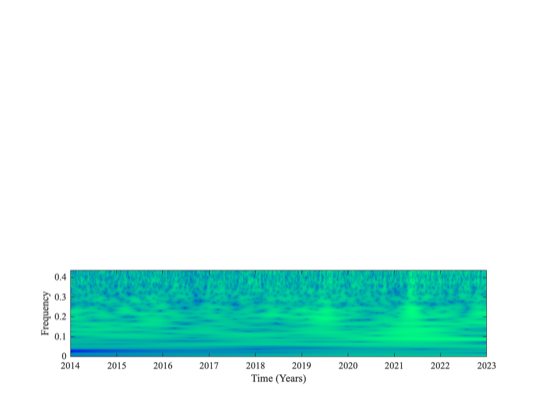 |
| ILARSP4=TA | SAIC | SAABBs |
| 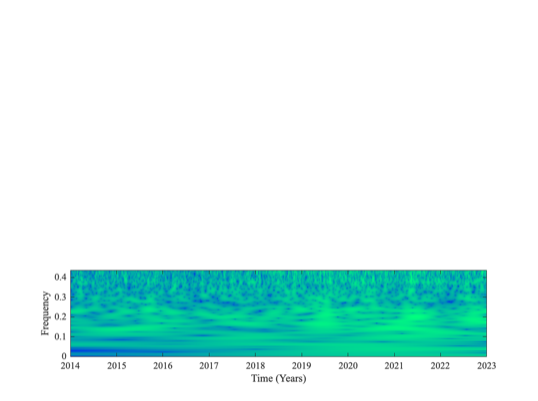 | 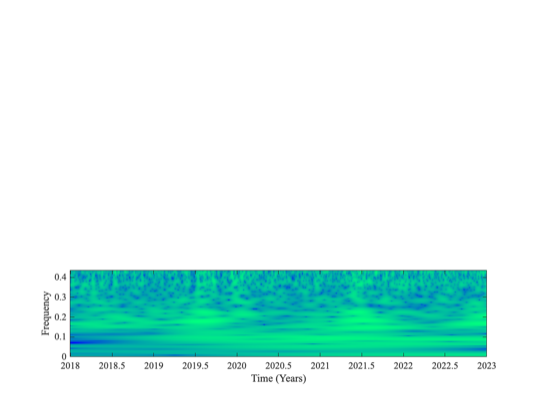 | 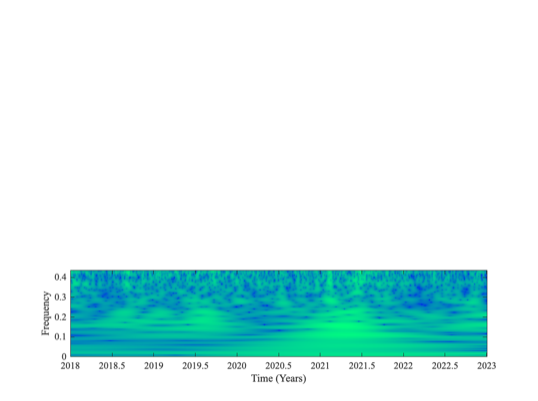 |
| BAB | HIAE | RFL |
| 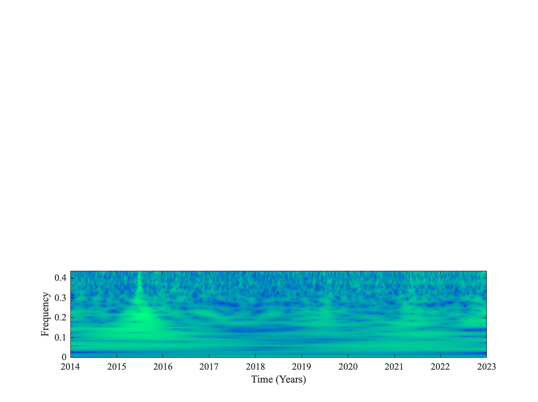 | 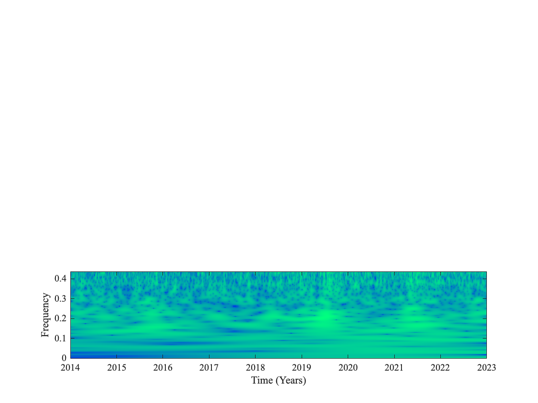 | 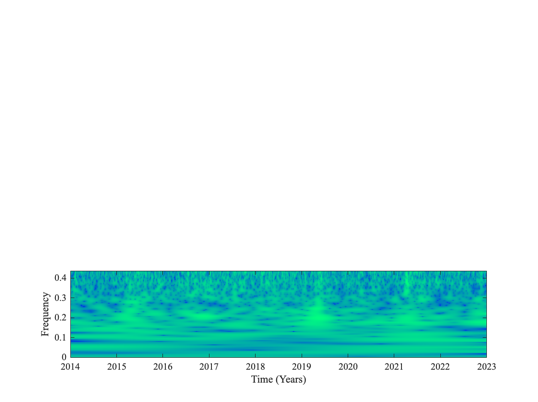 |
| 7011 | TXT | FCT |
| 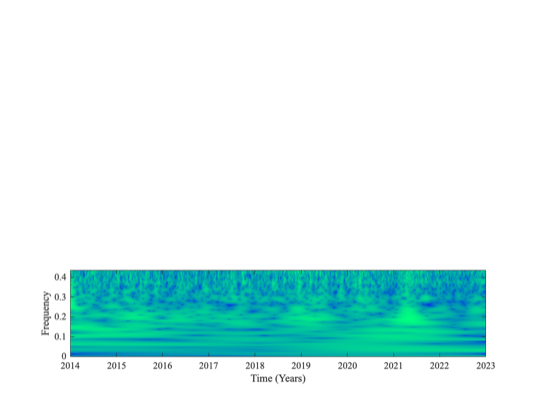 | 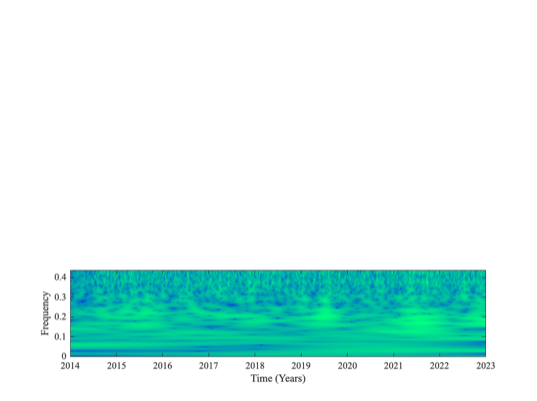 | 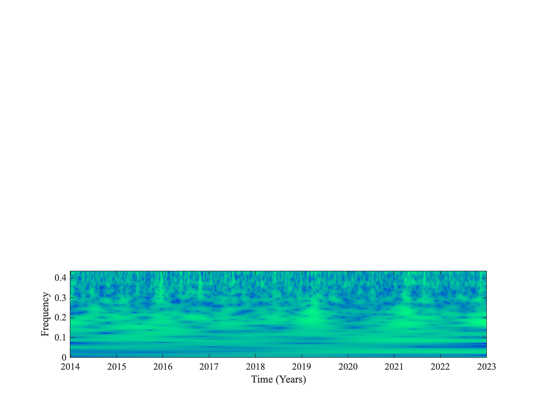 |
| CEAD | 012450 | VVX |
| 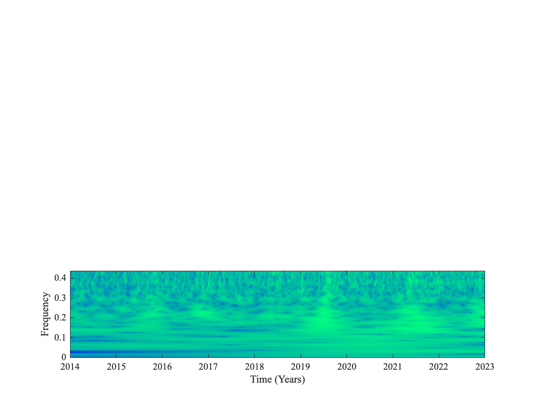 | 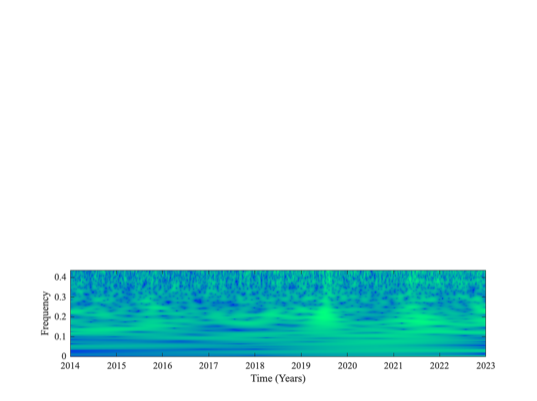 | 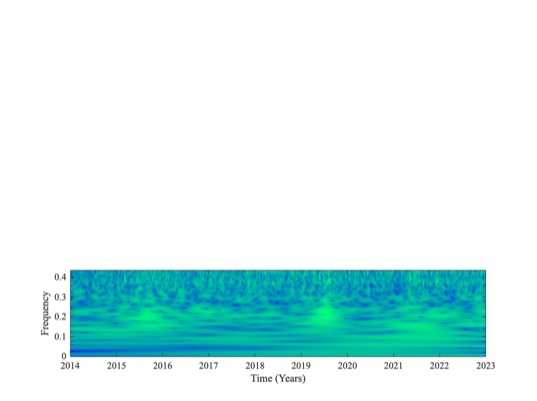 |
| TDG | PH | STEG |
| 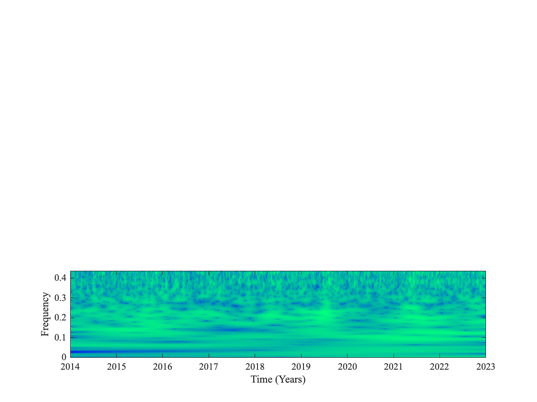 | 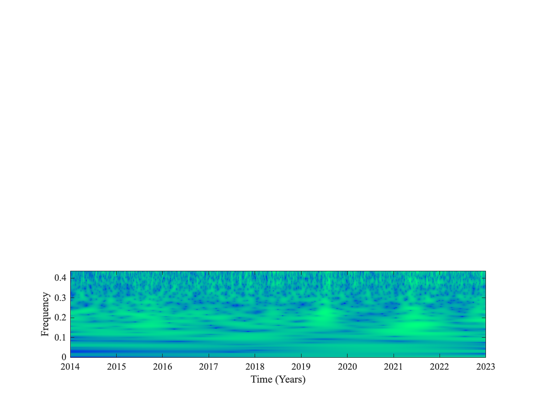 | 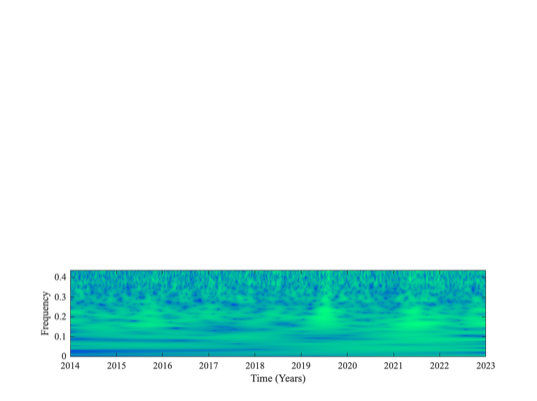 |
| OSK | J | TDY |
| 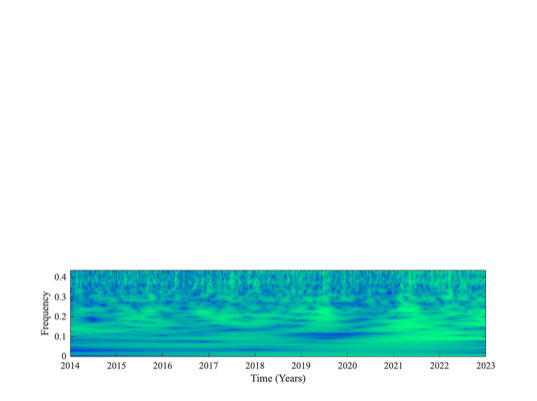 | 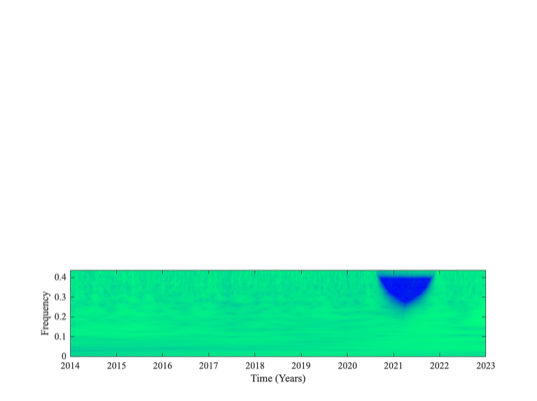 | 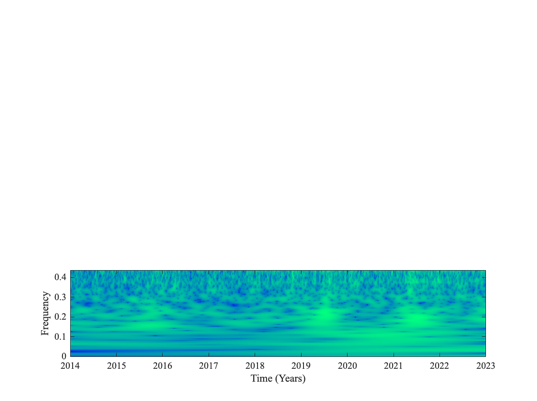 |
| ASELS | 2302 | TKAG |
| 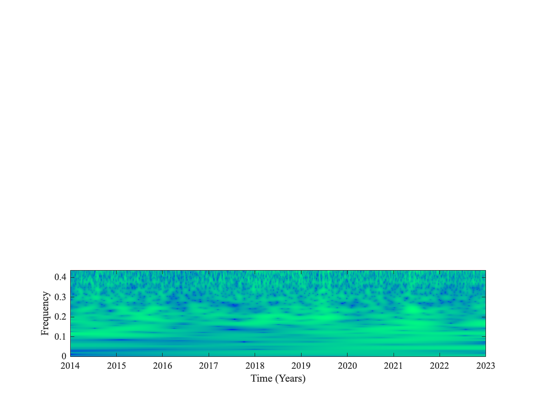 | 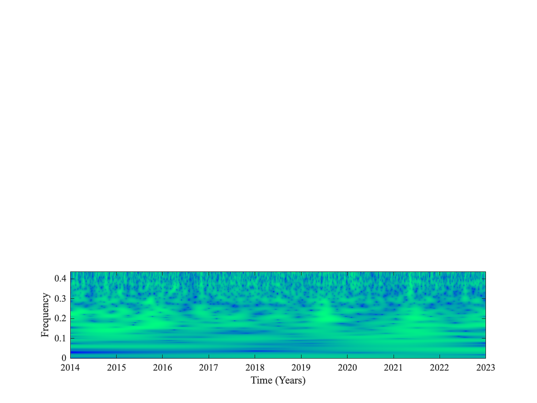 | 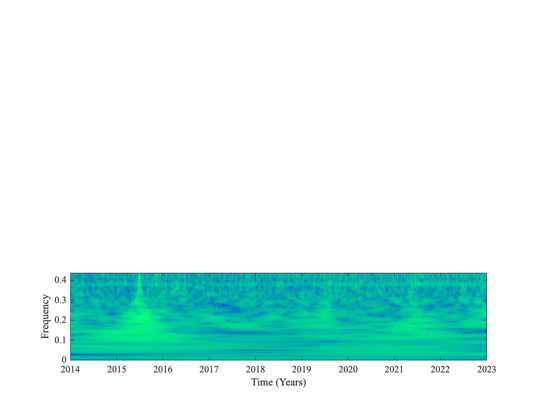 |
| BAJE | SRP | 7012 |
| 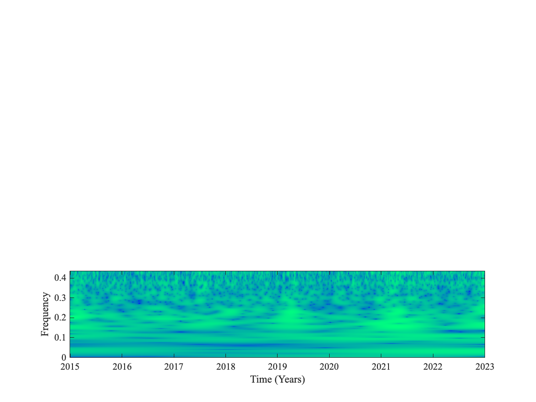 | 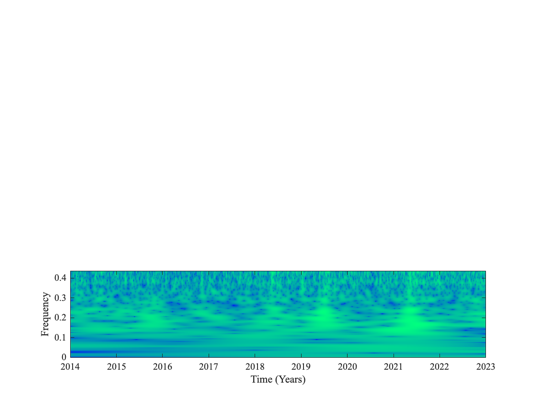 | 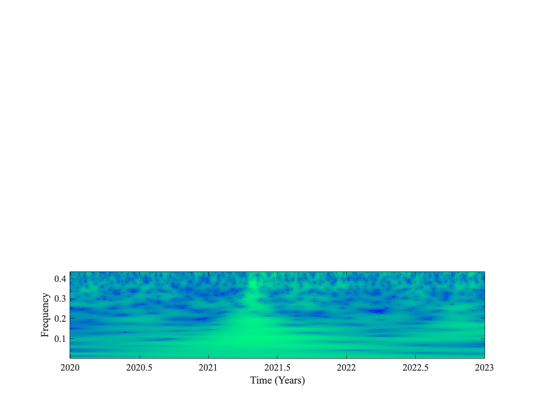 |
| 079550 | BWXT | HAGG |
| 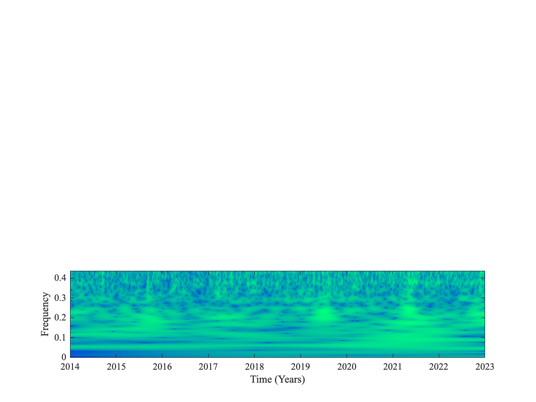 | 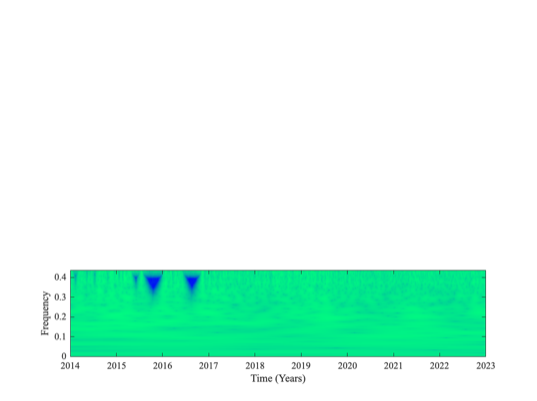 | 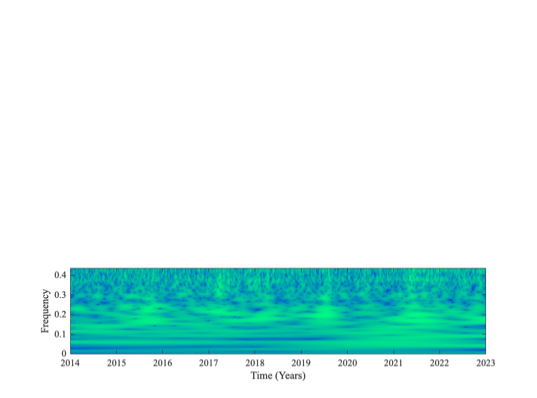 |
| QQ | PGZ | 047810 |
| 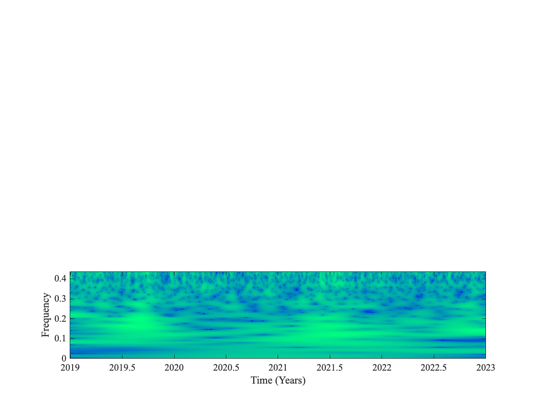 | 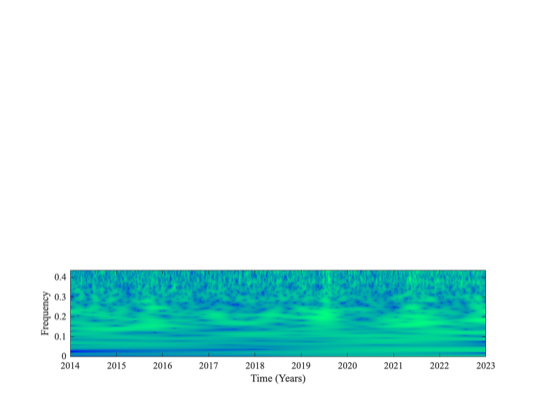 | 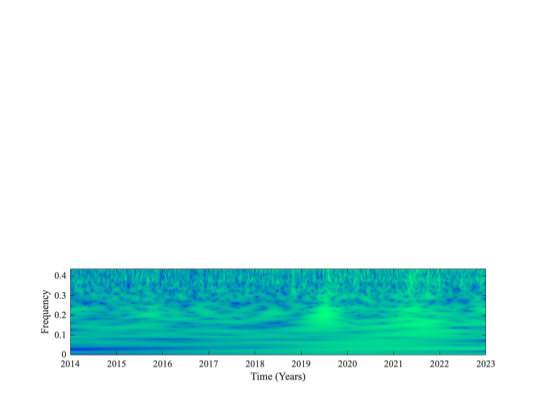 |
| PSN | ETN | CAE |
| 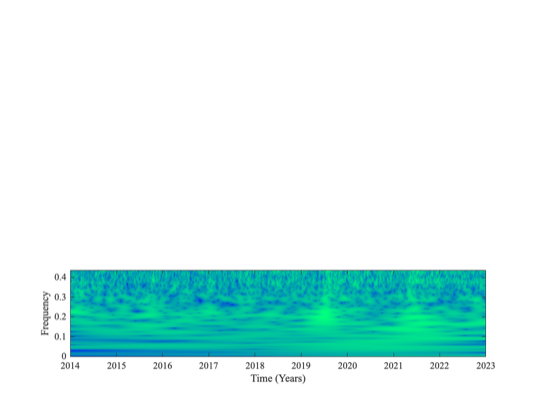 | 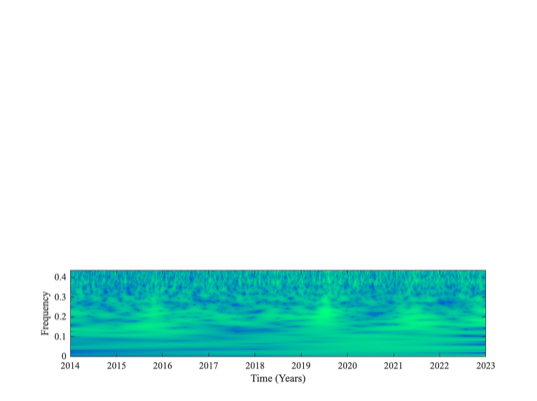 | 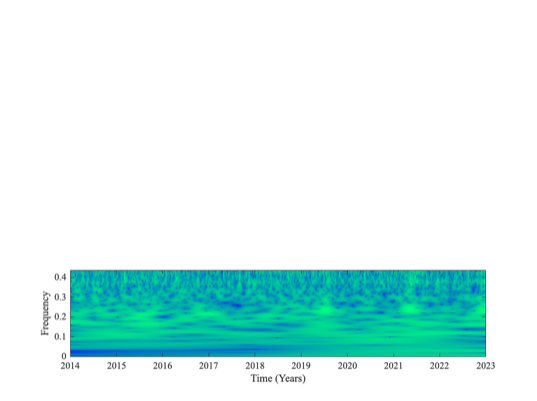 |
| CW | MOGa | 6755 |
| 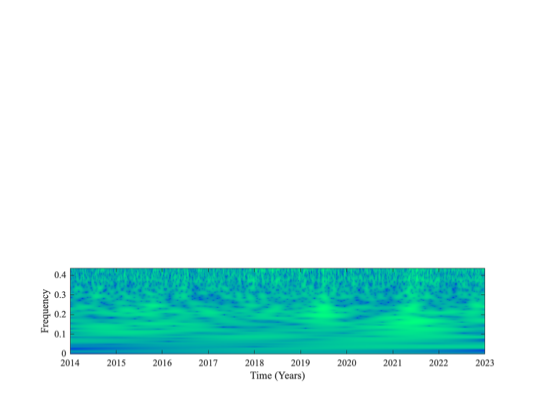 | 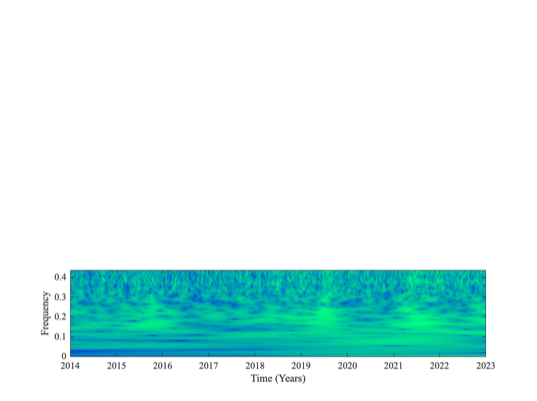 | 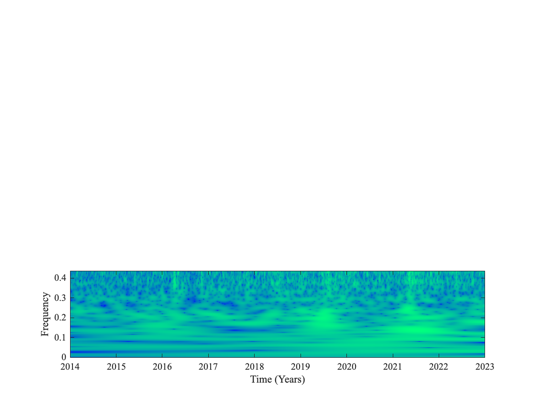 |
| KOG | APH | MRON |
| 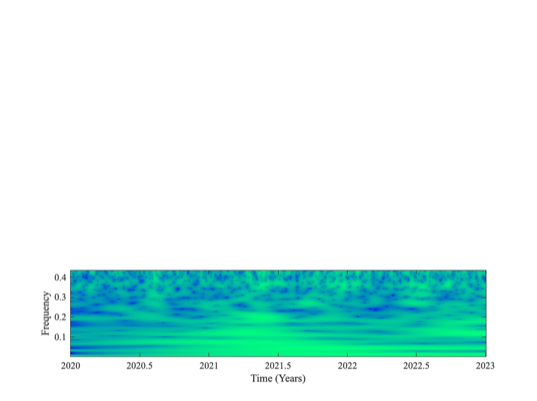 | 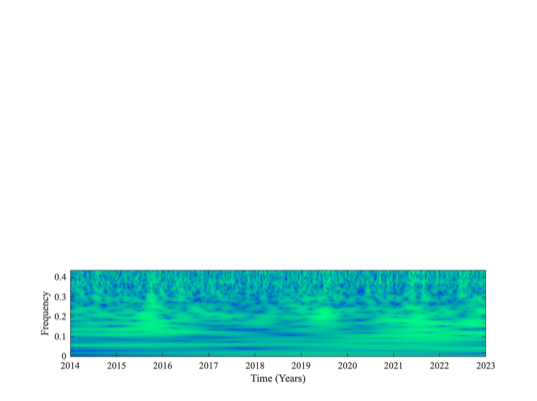 | 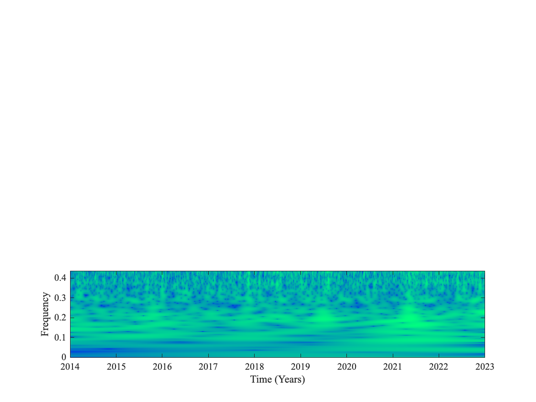 |
| MAZG | ASB | MRCY |
| 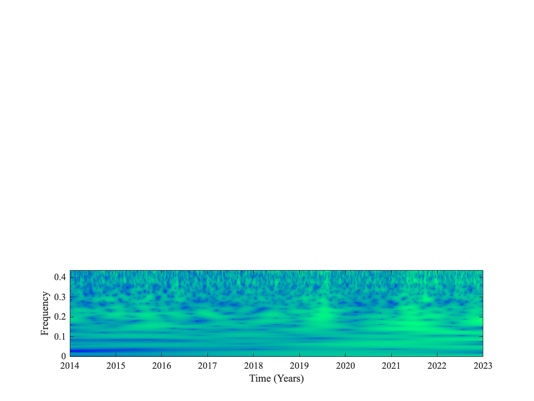 | 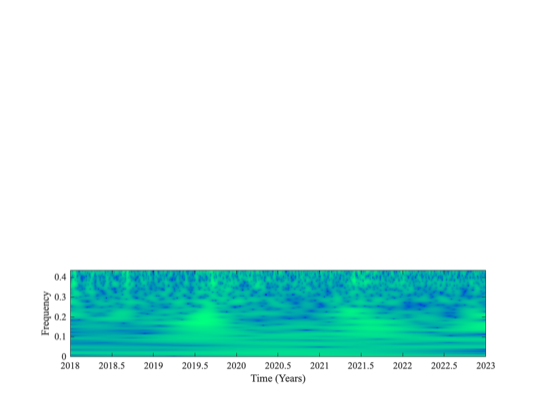 | 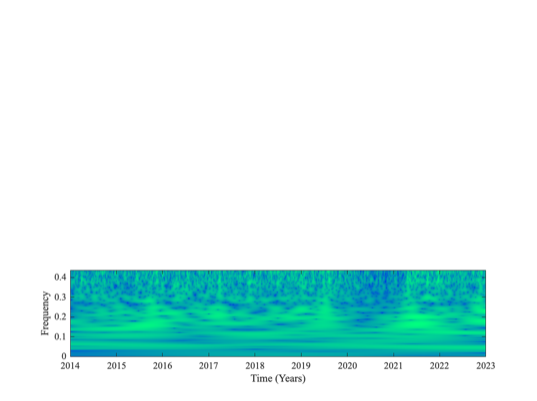 |
| BALL | HWM | TTMI |
| 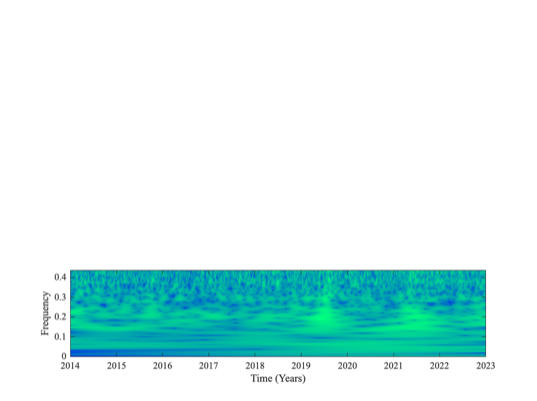 | 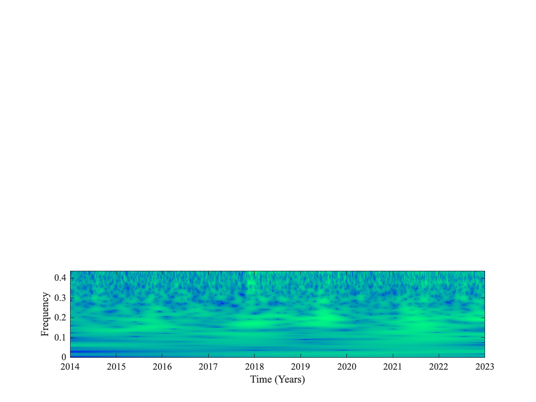 | 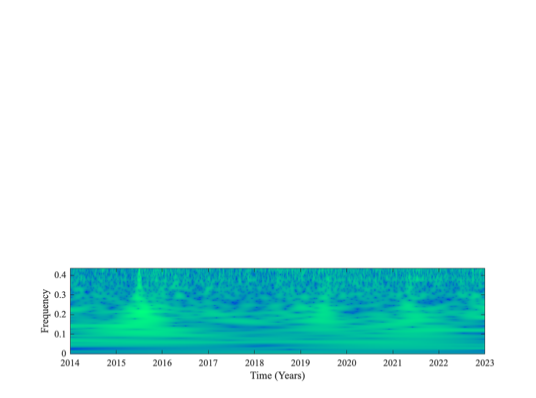 |
| HEI | 064350 | 7013 |

Note: Appendix 5 presents the Cross-Wavelet Transform (XWT) results, highlighting the time-frequency relationship between the Geopolitical Risk (GPR) index and the stock returns of the analyzed companies. The analysis reveals regions of significant comovement, capturing the impact of GPR on defense stocks over different time scales. Source: Authors’ computations.
