# Supplementary material for: Investigating the effect of geopolitical risk on defense companies’ stock returns
Source: Heliyon. 2024 Dec 7;10(24):e40974. doi: 10.1016/j.heliyon.2024.e40974 (PMC11700249; doi:10.1016/j.heliyon.2024.e40974)
Supplement: Multimedia component 6 [file mmc6.docx]

Appendix 6

Time series stability analysis for selected companies – structural break tests

| Company | Zivot-Andrews | | Dickey-Fuller min-t | | | Phillips-Perron | | ARCH | ARCH |
| --- | --- | --- | --- | --- | --- | --- | --- | --- | --- |
|  | t-Stat. | Break | t-Stat. | Prob. | Break | Adj.  t-Stat. | Prob. | LM-Stat. | Prob. |
| LMT | -2.890 | 22-Jan-2014 | -51.461 | <0.01 | Jan-2014 | -51.498 | 0.0001 | 576.70 | 0.000 |
| RYTT34 | -3.465 | 20-Feb-2015 | -50.001 | <0.01 | Jan-2015 | -49.431 | 0.0001 | 57.047 | 0.000 |
| NOC | -1.763 | 08-Jul-2016 | 53.534 | <0.01 | Jan-2014 | -54.145 | 0.0001 | 510.53 | 0.000 |
| BA | -3.548 | 16-Feb-2017 | -47.851 | <0.01 | Jan-2014 | -47.922 | 0.0001 | 854.86 | 0.000 |
| GD | -2.433 | 08-Jul-2016 | -51.243 | <0.01 | Jan-2014 | -51.175 | 0.0001 | 633.90 | 0.000 |
| BAES | -2.745 | 05-Feb-2014 | -49.072 | <0.01 | Jan-2014 | -49.040 | 0.0001 | 335.49 | 0.000 |
| 000065 | -2.217 | 21-Apr-2016 | -46.998 | <0.01 | Apr-2015 | -44.086 | 0.0001 | 15.717 | 0.046 |
| 000768 | -4.193 | 14-Nov-2014 | -46.748 | <0.01 | Jan-2014 | -46.715 | 0.0001 | 432.48 | 0.000 |
| 600879 | -5.361 | 20-Nov-2014 | -49.569 | <0.01 | Jan-2014 | -49.597 | 0.0001 | 1.7780 | 0.987 |
| 002268 | -5.167 | 10-Nov-2014 | -47.839 | <0.01 | Jan-2014 | -47.919 | 0.0001 | 7.2580 | 0.509 |
| LHX | -2.507 | 05-Feb-2014 | -53.098 | <0.01 | Jan-2014 | -53.183 | 0.0001 | 406.56 | 0.000 |
| LDOF | -2.082 | 30-Jan-2015 | -49.185 | <0.01 | Jan-2014 | -49.138 | 0.0001 | 103.90 | 0.000 |
| AIR | -2.789 | 23-Feb-2017 | -48.154 | <0.01 | Jan-2014 | -48.072 | 0.0001 | 723.26 | 0.000 |
| 600685 | -3.422 | 05-Nov-2014 | -45.673 | <0.01 | Jan-2014 | -45.836 | 0.0001 | 440.80 | 0.000 |
| TCPF | -2.105 | 16-Feb-2017 | -48.512 | <0.01 | Jan-2014 | -48.450 | 0.0001 | 394.86 | 0.000 |
| HII | -2.480 | 21-Oct-2014 | -51.787 | <0.01 | Mar-2020 | -51.192 | 0.0001 | 321.81 | 0.000 |
| LDOS | -3.133 | 24-Feb-2014 | -51472 | <0.01 | Mar-2020 | -51.340 | 0.0001 | 92.866 | 0.000 |
| BAH | -2.385 | 26-Sep-2014 | -53.529 | <0.01 | May-2017 | -51.702 | 0.0001 | 47.069 | 0.000 |
| AM | -2.927 | 16-Feb-2017 | -51.548 | <0.01 | Feb-2020 | -50.834 | 0.0001 | 227.98 | 0.000 |
| ESLT | -1.662 | 11-Mar-2016 | -54.186 | <0.01 | Jan-2014 | -54.230 | 0.0001 | 100.88 | 0.000 |
| RR | -2.294 | 13-Jun-2016 | -49.174 | <0.01 | Jan-2014 | -49.162 | 0.0001 | 0.1640 | 1.000 |
| CACI | -2.065 | 21-Oct-2014 | -54.677 | <0.01 | Jan-2014 | -54.490 | 0.0001 | 393.95 | 0.000 |
| HON | -2.359 | 17-Feb-2017 | -52.549 | <0.01 | Jan-2014 | -52.615 | 0.0001 | 892.72 | 0.000 |
| RHMG | -2.577 | 16-Feb-2017 | -46.641 | <0.01 | Feb-2020 | -46.035 | 0.0001 | 251.90 | 0.000 |
| GE | -1.986 | 25-Feb-2016 | -51.239 | <0.01 | Jan-2014 | -51.207 | 0.0001 | 469.46 | 0.000 |
| KBR | -2.934 | 10-Feb-2014 | -52.959 | <0.01 | Jan-2014 | -52.879 | 0.0001 | 655.99 | 0.000 |
| SAF | -2.749 | 16-Feb-2017 | -48.480 | <0.01 | Jan-2014 | -48.705 | 0.0001 | 1035.8 | 0.000 |
| ILARSP4=TA | -3.612 | 07-Jul-2015 | -53.436 | <0.01 | May-2015 | -53.833 | 0.0001 | 90.728 | 0.000 |
| SAIC | -2.098 | 21-Nov-2014 | -54.095 | <0.01 | Jan-2014 | -53.967 | 0.0001 | 255.43 | 0.000 |
| SAABBs | -2.081 | 20-Feb-2017 | -48.622 | <0.01 | Jan-2014 | -48.669 | 0.0001 | 137.71 | 0.000 |
| BAB | -1.176 | 10-Feb-2016 | -50.753 | <0.01 | Mar-2021 | -48.617 | 0.0001 | 24.992 | 0.001 |
| HIAE | -2.277 | 23-Jul-2018 | -36.608 | <0.01 | Aug-2020 | -36.013 | 0.0001 | 52.359 | 0.000 |
| RFL | -3.334 | 30-Mar-2020 | -49.092 | <0.01 | Oct-2021 | -38.039 | 0.0000 | 0.0210 | 1.000 |
| 7011 | -3.699 | 01-Sep-2015 | -134.433 | <0.01 | Sep-2015 | -51.073 | 0.0001 | 0.0020 | 1.000 |
| TXT | -2.677 | 17-Feb-2017 | -51.966 | <0.01 | Feb-2014 | -57.917 | 0.0001 | 597.72 | 0.000 |
| FCT | -2.886 | 22-May-2017 | -46.297 | <0.01 | Feb-2020 | -45.452 | 0.0001 | 151.46 | 0.000 |
| CEAD | -2.540 | 11-Apr-2014 | -53.460 | <0.01 | Jan-2014 | -51.481 | 0.0001 | 79.521 | 0.000 |
| 012450 | -2.119 | 04-Feb-2014 | -50.372 | <0.01 | Feb-2014 | -50.510 | 0.0001 | 35.067 | 0.000 |
| VVX | -2.343 | 08-Nov-2016 | -57.821 | <0.01 | Nov-2016 | -53.057 | 0.0001 | 1.9490 | 0.982 |
| TDG | -1.841 | 04-Feb-2014 | -51.800 | <0.01 | Feb-2014 | -51.773 | 0.0001 | 621.82 | 0.000 |
| PH | -2.071 | 17-Feb-2017 | -52.357 | <0.01 | Feb-2014 | -52.275 | 0.0001 | 845.48 | 0.000 |
| STEG | -1.958 | 17-Dec-2014 | -54.377 | <0.01 | Feb-2014 | -54.220 | 0.0001 | 409.45 | 0.000 |
| OSK | -2.456 | 13-Jun-2016 | -51.338 | <0.01 | Feb-2014 | -51.200 | 0.0001 | 116.68 | 0.000 |
| J | -1.869 | 22-Jan-2014 | -50.839 | <0.01 | Jan-2014 | -50.965 | 0.0001 | 337.51 | 0.000 |
| TDY | -1.831 | 19-Jan-2015 | -53.231 | <0.01 | Feb-2014 | -53.155 | 0.0001 | 442.55 | 0.000 |
| ASELS | -2.501 | 17-Oct-2016 | -56.326 | <0.01 | Feb-2023 | -53.583 | 0.0001 | 551.48 | 0.000 |
| 2302 | -1.902 | 27-Feb-2014 | -50.706 | <0.01 | Feb-2014 | -50.607 | 0.0001 | 264.77 | 0.000 |
| TKAG | -2.401 | 15-Feb-2017 | -48.955 | <0.01 | Mar-2020 | -48.189 | 0.0001 | 180.58 | 0.000 |
| BAJE | -2.999 | 09-May-2014 | -51.516 | <0.01 | Sep-2018 | -50.975 | 0.0001 | 171.07 | 0.000 |
| SRP | -2.965 | 25-Jun-2014 | -52.411 | <0.01 | Nov-2014 | -49.150 | 0.0001 | 5.8170 | 0.667 |
| 7012 | -3.438 | 01-Sep-2015 | -51.032 | <0.01 | Feb-2016 | -50.942 | 0.0001 | 0.0030 | 1.000 |
| 079550 | -3.978 | 16-Nov-2015 | -45.867 | <0.01 | Nov-2015 | -45.677 | 0.0001 | 21.577 | 0.005 |
| BWXT | -2.753 | 08-Jul-2016 | -50.966 | <0.01 | Feb-2014 | -50.917 | 0.0001 | 21.472 | 0.006 |
| HAGG | -2.060 | 17-Feb-2022 | -24.440 | <0.01 | Oct-2020 | -24.107 | 0.0000 | 130.41 | 0.000 |
| QQ | -1.858 | 28-Jan-2014 | -53.101 | <0.01 | Dec-2020 | -52.321 | 0.0001 | 45.639 | 0.000 |
| PGZ | -2.585 | 08-Apr-2016 | -56.294 | <0.01 | Feb-2014 | -61.469 | 0.0001 | 75.691 | 0.000 |
| 047810 | -2.819 | 21-Jan-2015 | -52.855 | <0.01 | Oct-2018 | -51.724 | 0.0001 | 26.328 | 0.001 |
| PSN | -4.579 | 22-Oct-2019 | -36.526 | <0.01 | May-2019 | -36.476 | 0.0000 | 144.60 | 0.000 |
| ETN | -2.164 | 28-Jan-2014 | -53.519 | <0.01 | Feb-2014 | -53.480 | 0.0001 | 667.57 | 0.000 |
| CAE | -2.173 | 17-Feb-2017 | -48.997 | <0.01 | Feb-2014 | -49.034 | 0.0001 | 547.68 | 0.000 |
| CW | -2.240 | 17-Feb-2014 | -52.160 | <0.01 | Feb-2014 | -51.986 | 0.0001 | 569.64 | 0.000 |
| MOGa | -2.479 | 17-Feb-2017 | -51.955 | <0.01 | Feb-2014 | -51.801 | 0.0001 | 535.61 | 0.000 |
| 6755 | -2.366 | 26-Mar-2014 | -49.835 | <0.01 | Jan-2021 | -49.159 | 0.0001 | 27.967 | 0.001 |
| KOG | -1.520 | 07-Feb-2014 | -55.002 | <0.01 | Apr-2022 | -53.418 | 0.0001 | 18.519 | 0.017 |
| APH | -1.983 | 17-Feb-2017 | -53.236 | <0.01 | Feb-2014 | -53.172 | 0.0001 | 514.70 | 0.000 |
| MRON | -3.110 | 10-Jun-2016 | -51.071 | <0.01 | Feb-2014 | -51.177 | 0.0001 | 99.056 | 0.000 |
| MAZG | -1.601 | 14-Dec-2020 | -28.244 | <0.01 | Oct-2020 | -28.142 | 0.0000 | 35.656 | 0.000 |
| ASB | -3.192 | 19-Jan-2015 | -53.757 | <0.01 | Dec-2015 | -52.332 | 0.0001 | 20.097 | 0.010 |
| MRCY | -3.046 | 14-Mar-2014 | -52.386 | <0.01 | Feb-2014 | -52.375 | 0.0001 | 116.42 | 0.000 |
| BALL | -2.669 | 05-Jun-2018 | -50.977 | <0.01 | Feb-2014 | -51.215 | 0.0001 | 113.27 | 0.000 |
| HWM | -6.510 | 27-Apr-2018 | -39.878 | <0.01 | Apr-2018 | -38.965 | 0.0000 | 210.19 | 0.000 |
| TTMI | -3.576 | 07-Jul-2016 | -55.290 | <0.01 | Aug-2017 | -54.289 | 0.0001 | 60.477 | 0.000 |
| HEI | -2.441 | 17-Feb-2017 | -50.869 | <0.01 | Feb-2014 | -50.769 | 0.0001 | 675.12 | 0.000 |
| 064350 | -2.623 | 05-Feb-2014 | -54.518 | <0.01 | Feb-2014 | -54.393 | 0.0001 | 245.43 | 0.000 |
| 7013 | -2.911 | 31-Aug-2015 | -102.425 | <0.01 | Aug-2015 | -50.405 | 0.0001 | 0.0030 | 1.000 |

Note: Appendix 6 presents results from the Zivot-Andrews, Dickey-Fuller, Phillips-Perron, and ARCH tests. The Zivot-Andrews, Dickey-Fuller, and Phillips-Perron tests assess stationarity, with the Zivot-Andrew’s test incorporating structural breaks. The ARCH LM test evaluates conditional heteroskedasticity in stock returns. Source: Author’s computation based on historical daily returns.
